# Cell-intrinsic steroidogenesis regulates mast cell function

**DOI:** 10.1126/sciadv.aea0260

**Published:** 2026-10-16

**Authors:** Jhuma Pramanik, Qiuchen Zhao, Soura Chakraborty, Yumi Yamashita-Kanemaru, Hosni Hussein, Natalie Z. M. Homer, Sanu K. Shaji, Silia Ayadi, Mohsen Ali Asgari, William J. Griffiths, Yuqin Wang, Klaus Okkenhaug, Rahul Roychoudhuri, Bidesh Mahata

**Affiliations:** ^1^ Department of Pathology, University of Cambridge, Cambridge CB2 1QP, UK.; ^2^ Mass Spectrometry Core, Edinburgh Clinical Research Facility, Queens Medical Research Institute, University of Edinburgh, Edinburgh, UK.; ^3^ Swansea University Medical School, ILS1 Building, Singleton Park, Swansea SA2 8PP, UK.

## Abstract

Steroid hormones are well-known regulators of mast cell function, but whether mast cells themselves produce steroids de novo and influences their effector function has remained unknown. Here, we show that mast cells constitutively express the steroidogenic enzyme cytochrome P450 family 11 subfamily A member 1 (Cyp11a1) and produce pregnenolone. Cyp11a1 expression was detected across tissue-resident mast cells from multiple organs, was conserved in human mast cells, and was enhanced by interleukin-3 signaling and after activation. We identified Cyp11a1 as a Gata2-dependent, superenhancer-associated mast cell gene, placing steroidogenesis within the core mast cell transcriptional program. Loss of Cyp11a1 altered transcriptomic and proteomic programs in mast cells, with effects on pathways linked to cell growth, immune signaling, and effector function. Functionally, Cyp11a1-deficient mast cells showed increased degranulation in response to both immunoglobulin E (IgE)–dependent and IgE-independent stimulation. In vivo, loss of Cyp11a1 reduced mast cell abundance in several tissues but exacerbated passive cutaneous anaphylaxis. These findings identify cell-intrinsic steroidogenesis as a regulatory pathway that restrains mast cell activation and anaphylactic responses.

## INTRODUCTION

Mast cells are tissue-resident immune cells of hematopoietic origin that are strategically positioned at barrier surfaces throughout the body (*1*–*6*). These cells contribute to host defense, vascular homeostasis, and tissue remodeling, but their dysregulation underlies allergic and inflammatory pathologies (*1*, *4*, *6*). Two major subtypes have been identified in rodents and humans: connective tissue mast cells and mucosal mast cells, each characterized by distinct protease and mediator profiles (*1*–*6*). Upon activation, mast cells rapidly release their stored granule contents, followed by the synthesis and secretion of pro-inflammatory lipid mediators, chemokines, and cytokines (*7*). Following degranulation, mast cells enter a postdegranulation recovery phase in which they restore secretory granule content and undergo substantial transcriptional and translational remodeling (*8*–*10*). The cellular and molecular events governing this phase, and how they shape subsequent mast cell responsiveness and tissue homeostasis, remain poorly understood.

Despite their critical protective physiological functions, dysregulation of mast cells can lead to various pathological conditions, including allergic and inflammatory disorders. Multiple regulatory mechanisms modulate mast cell activation. These include inhibitory receptors containing immunoreceptor tyrosine-based inhibitory motifs (*11*, *12*), as well as innate signaling pathways that operate independently of immunoglobulin E (IgE) (*13*). How mast cells restrain their effector function and reestablish tissue homeostasis during this recovery phase remains poorly understood.

Steroidogenesis is the biosynthetic process by which steroid hormones are synthesized from cholesterol. Cytochrome P450 family 11 subfamily A member 1 (CYP11A1; also known as P450scc) catalyzes the first and rate-limiting step of this pathway, converting cholesterol to pregnenolone (Fig. 1A, left panel) (*14*, *15*). Pregnenolone is the first bioactive steroid hormone of the pathway, and it serves as the precursor of all other steroid hormones. Steroid glands, such as the adrenal gland, ovary, testis, and placenta, produce gland-specific steroid hormones to control several physiological processes in vertebrates, including salt balance, carbohydrate metabolism, sex differentiation, and immune regulation (*14*, *15*). The regulation of mast cell function by steroid hormones is well established, and synthetic steroids has been exploited therapeutically to treat allergic and inflammatory conditions (*16*–*18*). However, whether mast cells themselves have steroidogenic capacity and a cell-intrinsic steroid-regulatory mechanism has not been investigated.

**Fig. 1.**
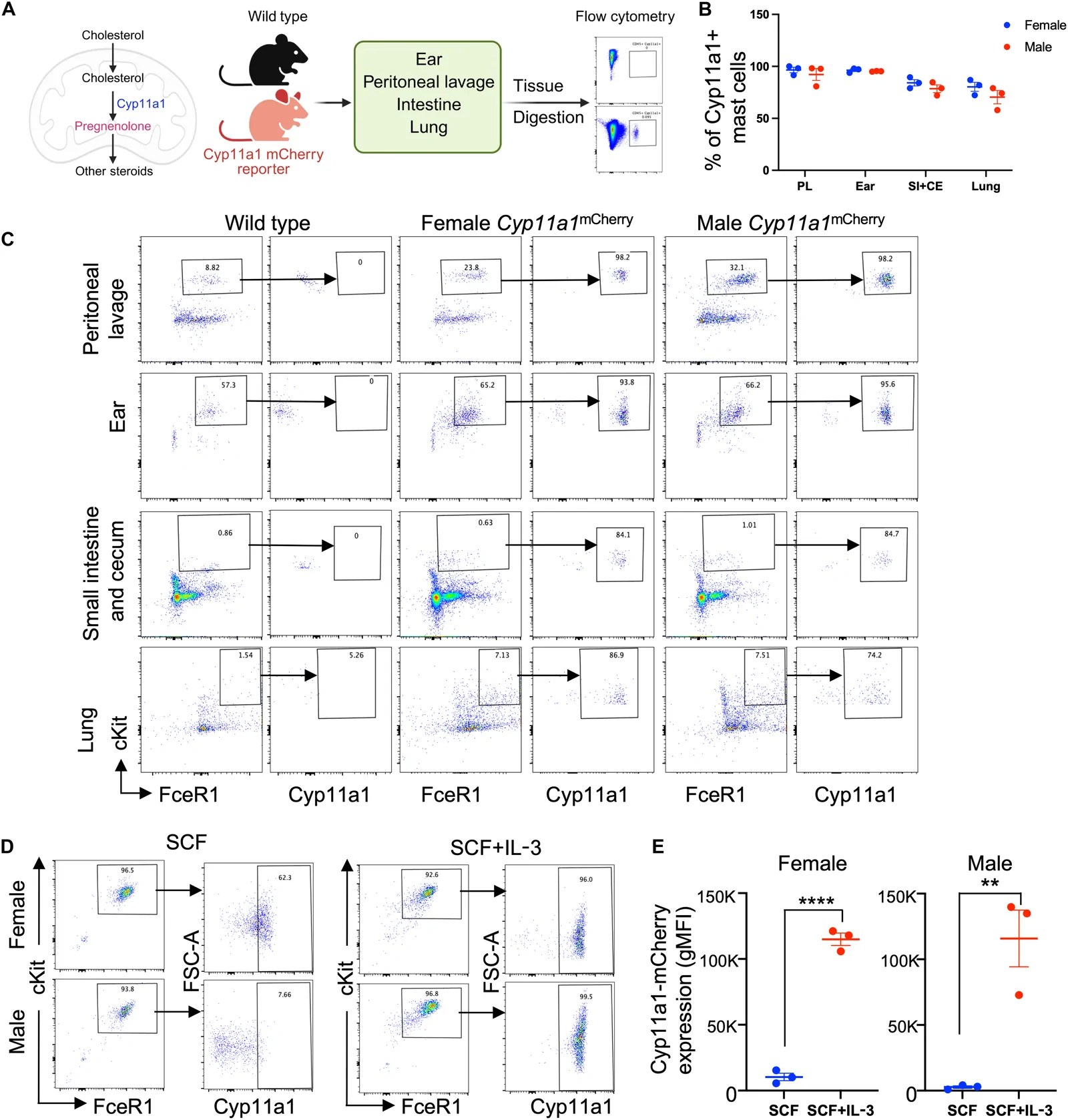
Cyp11a1 expression by mast cells. (**A**) Schematic representation of the Cyp11a1 activity catalyzing the rate-limiting step of steroidogenesis (schematic on the left) and experimental plan (schematic on the right) used in (B) and (C). Tissues and organs of healthy, unchallenged Cyp11a1-mCherry reporter or wild-type mice were mechanically dissociated and enzymatically digested. Single-cell suspensions were analyzed by flow cytometry to measure Cyp11a1 expression. Created in BioRender. B. Mahata (2026), https://BioRender.com/uexu9p0 and https://BioRender.com/0nxkxsk. (**B**) Graphical presentation of the Cyp11a1-mCherry expression in mast cells obtained from specified tissue origin. PL, peritoneal lavage; SI + CE, small intestine and cecum. Representative of three independent experiments. Each experiment contains three to six mice. (**C**) Representative fluorescence-activated cell sorting (FACS) profile of Cyp11a1-mCherry expression shown in (B). FACS gating: all cells > singlets > live cells > CD45^+^ > F4/80^−^ > ST2^+^ > cKit^+^FceR1^+^ > Cyp11a1-mCherry^+^. (**D** and **E**) Effect of IL-3 on Cyp11a1 expression in PMCs. (D) Cyp11a1-mCherry mouse–derived PMCs were propagated in vitro in the presence or absence of IL-3, and Cyp11a1-mCherry expression was detected by flow cytometry (representative FACS plots). (E) Graphical presentation of Cyp11a1-mCherry expression in female and male cohorts (*n* = 3). gMFI, geometric mean fluorescence intensity. The bars show the means ± SEM. The *P* value was calculated using an unpaired two-tailed *t* test (****P ≤ 0.0001, **P ≤ 0.01, and ns for *P* > 0.05).

Here, we report that mast cells express functional Cyp11a1 and constitutively produce the steroid pregnenolone. Using genetic, transcriptomic, proteomic, and functional approaches, we demonstrate that this cell-intrinsic steroidogenesis pathway shapes mast cell gene expression programs and acts as a negative regulator of inflammatory activation both in vitro and in vivo. In the absence of mast cell steroidogenesis, mice display markedly exaggerated anaphylactic responses, establishing Cyp11a1-dependent steroidogenesis as a homeostatic brake on mast cell function.

## RESULTS

### Mast cells express Cyp11a1

Whether mast cells have intrinsic steroidogenic capacity under homeostatic and inflammatory conditions has not been examined. We investigated Cyp11a1 expression in peripheral tissues and organs using Cyp11a1-mCherry reporter mice that have been described previously (Fig. 1A) (*19*). We found that tissue-resident mast cells in all examined tissues, namely, ear, peritoneum, intestine, lung, adrenal gland, and ovary, express Cyp11a1 to varying degrees (Fig. 1, B and C, and fig. S1A). To extend these observations, we analyzed publicly available single-cell transcriptomic datasets (*20*, *21*) and confirmed that *Cyp11a1* expression is detected in mast cells across diverse mouse tissues including gut mucosa, heart, mammary gland, muscle, skin, small intestine, uterus, and lung (fig. S1, B and D). Cyp11a1 expression is conserved in bone marrow–derived mast cells (BMMCs) generated in vitro from bone marrow precursors, as well as in peritoneal cavity–derived mast cells (fig. S1C). Reanalysis of publicly available bulk RNA sequencing (RNA-seq) data shows that *CYP11A1* expression is conserved in human mast cells, particularly in the skin (fig. S1E). In murine mast cells, interleukin-3 (IL-3) signaling increased Cyp11a1 expression (Fig. 1, D and E), whereas addition of adenosine 3′,5′-monophosphate did not further augment this response (fig. S1F). These findings indicate that Cyp11a1 expression is a conserved feature of mast cells.

### Cyp11a1 catalyzes pregnenolone biosynthesis

To determine whether Cyp11a1 expression results in functional steroid production, we differentiated BMMCs and peritoneal mast cells (PMCs) from Cyp11a1-sufficient (*Cyp11a1*^fl/fl^, Ctrl mice) (*19*) and Cyp11a1-deficient (*Cyp11a1*^fl/fl^;*Vav1*^Cre^ cKO) mice (Fig. 2A). *Cyp11a1* deletion efficiency has been shown in fig. S2. *Cyp11a1* deletion did not prevent mast cell differentiation but resulted in a statistically significant reduction in the proportion of cKit^+^FceRI^+^ mature mast cells in both BMMC and PMC cultures (Fig. 2B).

**Fig. 2.**
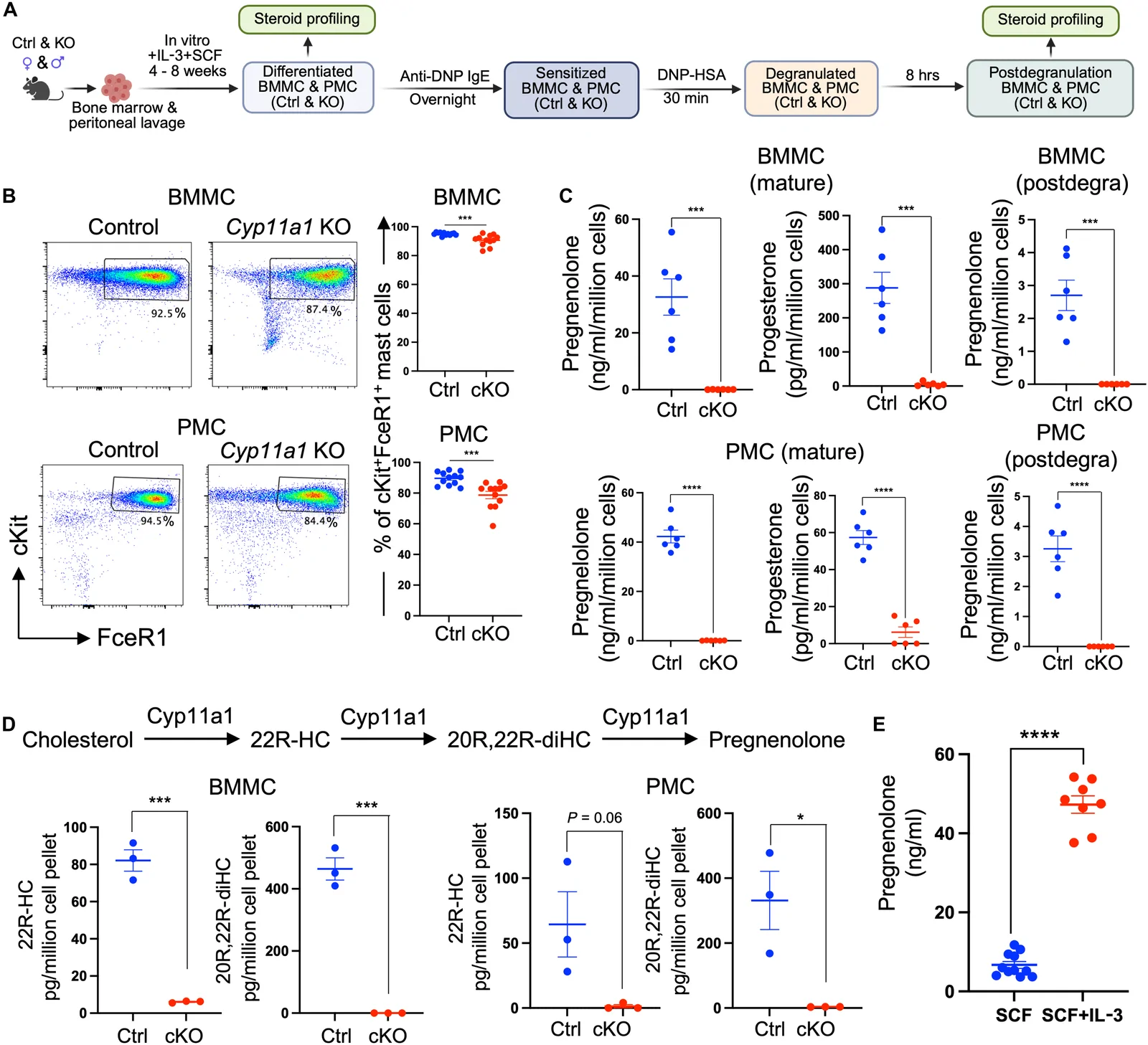
Mast cells synthesize steroid pregnenolone. (**A**) Schematic representation of the experimental plan. *Cyp11a1*^fl/fl^ (Ctrl) or *Cyp11a1* ^fl/fl^ *Vav1*^Cre^ (KO) mice’s bone marrow or peritoneal cavity–derived mast cells (BMMCs and PMCs) were in vitro–differentiated and activated. Eight hours after degranulation, cell culture supernatants of mature and postdegranulation mast cells were analyzed by LC-MS/MS for the presence of steroid hormones. Created in BioRender. B. Mahata (2026), https://BioRender.com/t40u919. (**B**) Effect of *Cyp11a1* KO in BMMC and PMC maturation (as defined by cKit and FceR1 expression). Left panel: Representative FACS plot. Right panel: Graphical presentation of cKit^+^FceR1^+^ cells (*n* = 12). (**C**) Steroid profiling of mature and postdegranulation BMMC and PMC culture supernatant. The presence of all major steroids was analyzed by LC-MS/MS. Only those steroids that show Cyp11a1-dependent synthesis, pregnenolone and progesterone, are displayed. Supernatants from mature (resting) mast cells were collected after 4 days of standard culture conditions (without stimulation). Supernatants from postdegranulation mast cells were collected 8 hours after IgE-mediated stimulation with DNP-HSA (*n* = 6). (**D**) Quantitative profiling of oxysterol, 22R-HC, and 20R,22R-diHC in mature and postdegranulation BMMCs and PMCs. These are the two intermediate oxysterol products during pregnenolone synthesis (schematic is shown in the top panel). *Cyp11a1*-sufficient or *Cyp11a1*-deficient mature mast cells were cultured for 4 days, and postdegranulation mast cells were cultured for 8 hours (*n* = 3, biologically independent mouse-derived mast cells). Cell pellets of equal number of cells were used for sterol extraction and quantification. (**E**) Effect of IL-3 in pregnenolone biosynthesis by PMCs. PMCs were cultured in the presence or absence of IL-3, and pregnenolone concentration was measured by ELISA. The bars show the means ± SEM. The *P* value was calculated using an unpaired two-tailed *t* test (****P ≤ 0.0001, ***P ≤ 0.001, *P ≤ 0.05, and actual *P* value for *P* > 0.05).

Steroid profiling of culture supernatants by liquid chromatography–tandem mass spectrometry (LC-MS/MS), using a panel encompassing the major steroid classes, revealed that mature mast cells produce substantial quantities of pregnenolone and detectable levels of progesterone (Fig. 2C). Genetic deletion of *Cyp11a1* abolished production of both steroids, confirming active Cyp11a1-mediated catalysis of cholesterol to pregnenolone (Fig. 2C). Analysis of oxysterol intermediates [i.e., 22*R*-hydroxycholesterol (22R-HC) and 20*R*,22*R*-dihydroxycholesterol (20R,22R-diHC)] further confirmed enzymatic activity through the canonical three-step oxidative conversion pathway (Fig. 2D). These intermediates were abolished in Cyp11a1-deficient (cKO) cells (Fig. 2D). Consistent with the observation of IL-3–mediated Cyp11a1 up-regulation (Fig. 1, D and E), we observed increased pregnenolone production upon IL-3 treatment in PMCs (Fig. 2E). Together, these data demonstrate that mast cell Cyp11a1 is enzymatically active and required for pregnenolone biosynthesis.

### *Cyp11a1* is a Gata2-regulated mast cell identity gene

To define how Cyp11a1 is regulated during mast cell activation, we performed RNA-seq and quantitative proteomic profiling of BMMCs and PMCs before and 8 hours after IgE-mediated degranulation. The time point has been chosen on the basis of previous studies showing the initiation of transcriptional and translational changes during this period (*8*–*10*, *22*–*24*). We observed that *Cyp11a1* expression (Fig. 3, A and B) and steroidogenesis (Fig. 3C) were increased after activation/degranulation. To confirm whether it is the activation signal or the degranulation process that induces Cyp11a1 expression, we tested the effect of degranulation inhibition on Cyp11a1 expression. Although nocodazole significantly inhibited degranulation (fig. S3A), it did not alter Cyp11a1 expression (fig. S3B).

**Fig. 3.**
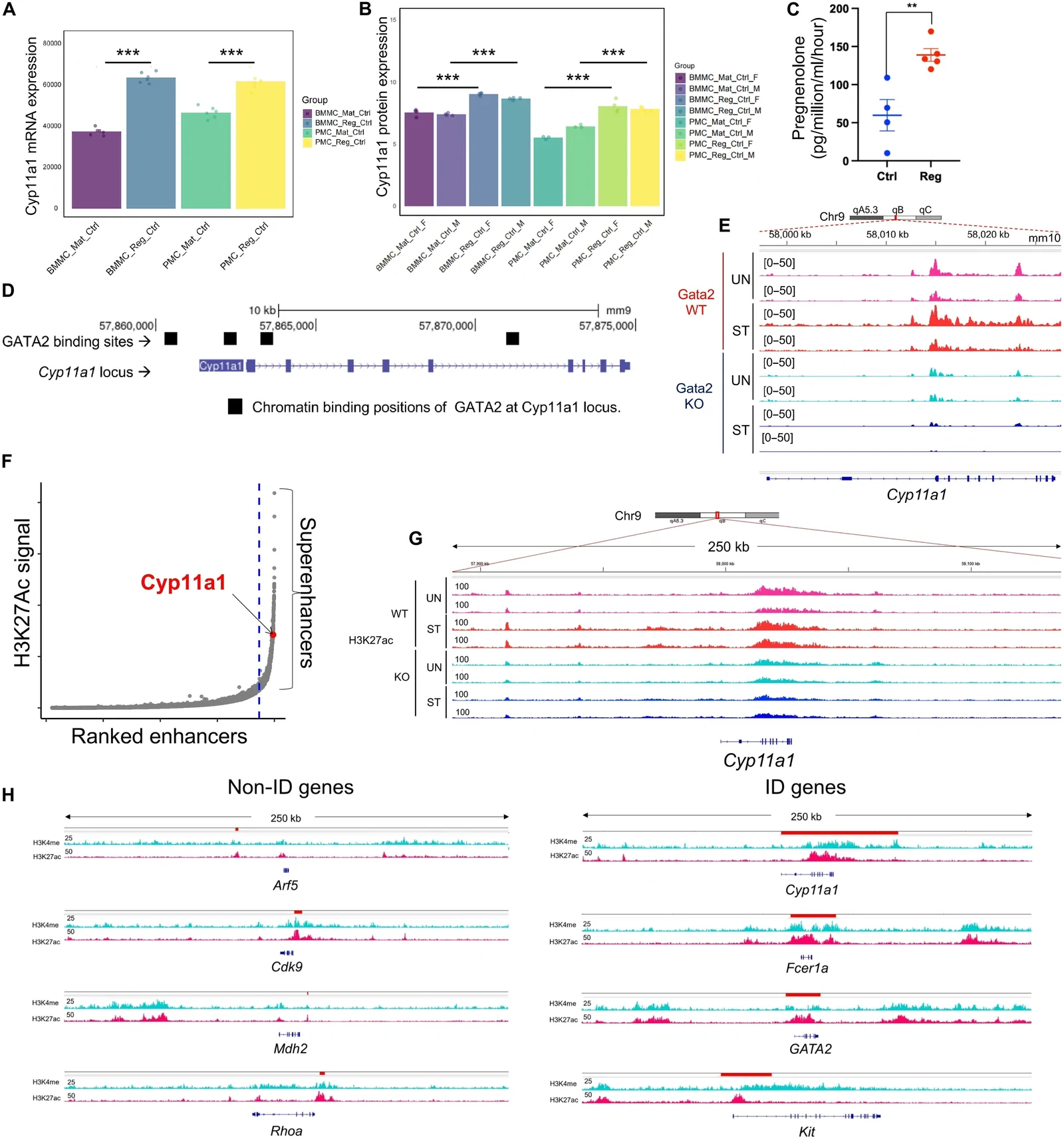
Regulation of Cyp11a1 locus in mast cells. (**A** and **B**) Mature BMMCs and PMCs were sensitized by an anti-DNP IgE antibody and activated by exposing them to DNP-HSA. Eight hours after degranulation, cells were analyzed by transcriptomics (RNA-seq) and proteomics (LC-MS/MS). Graphical representation of Cyp11a1 mRNA (A) and protein (B) expression. *P* values are calculated using pairwise *t* tests. ***P ≤ 0.001. (**C**) An equal number of mature and postdegranulation [regranulating (Reg)] PMCs were cultured, and supernatants were analyzed by pregnenolone ELISA (*n* = 5). Error bars: means ± SEM. The *P* value was calculated using an unpaired two-tailed *t* test (**P ≤ 0.01). (**D**) Genome Browser view showing the results of Gata2 ChIP-seq analysis at the *Cyp11a1* locus. Black boxes indicate Gata2 binding sites. (**E**) Gata2-dependent chromatin accessibility at the *Cyp11a1* locus in resting and IgE-activated BMMCs. Representative IGV tracks of Omni-ATAC-seq data at the *Cyp11a1* locus in wild-type (WT) and Gata2 KO mast cells under resting (UN) and stimulated (ST) conditions. (**F**) The Cyp11a1 locus is embedded within a mast cell superenhancer domain. Rank ordering of superenhancers (ROSE) analysis based on H3K27ac ChIP-seq signal in resting BMMCs. Each dot represents one enhancer element. Superenhancers are defined as those above the inflection point of the ranked H3K27ac signal curve. The Cyp11a1 superenhancer is highlighted, ranking 59th of 697 enhancers. (**G**) IgE receptor activation increases chromatin accessibility and H3K27ac modification at the *Cyp11a1* superenhancer domain. Representative IGV tracks showing Omni-ATAC-seq chromatin accessibility and H3K27ac ChIP-seq signal at the *Cyp11a1* locus in resting (UN) and IgE-activated (ST) BMMCs. (**H**) Representative IGV tracks of the Cyp11a1 and other representative mast cell identity (ID) genes and non-ID genes. The small bars below the non-ID gene tracks indicate typical enhancers, and the large bars below the ID genes indicate superenhancers. Data in (E) to (H) represent two biological replicates.

To investigate the transcriptional regulation of *Cyp11a1* in mast cells, we analyzed publicly available chromatin immunoprecipitation sequencing (ChIP-seq) data for the mast cell master regulator transcription factor (TF) Gata2 in primary mast cells and Omni-ATAC-seq datasets on BMMCs (*25*, *26*).This analysis revealed multiple GATA2 binding sites at the *Cyp11a1* locus, distributed both upstream of the transcription start site and within intronic regions (Fig. 3D). Four distinct GATA2 binding regions were identified across the locus at positions: chromosome 9 (chr9): 57860282 to 57860681, 57862123 to 57862522, 57863278 to 57863677, and 57870948 to 57871347 (mm9) (Fig. 3D). Analysis of Omni-ATAC-seq data from wild-type and *Gata2* knockout (KO) BMMCs (*25*) demonstrated that chromatin accessibility at the *Cyp11a1* locus was present in wild-type cells and was enhanced following IgE-mediated degranulation but was abolished in Gata2-deficient BMMCs (Fig. 3E). Together, these data indicate that Gata2 directly regulates *Cyp11a1* transcription in mast cells and that the *Cyp11a1* locus is dynamically regulated during the mast cell activation cycle. The Gata2 dependence of the Cyp11a1 locus prompted us to test whether Cyp11a1 has the regulatory features of a mast cell identity gene.

Superenhancers characterize the cell type–specific key identity genes (*27*–*29*). To assess whether the *Cyp11a1* locus is a mast cell identity gene and it is embedded within a superenhancer domain, we reanalyzed previously published rank ordering of superenhancers (ROSE) classification data generated from H3K27ac ChIP-seq in resting BMMCs (*25*). We found that the *Cyp11a1* locus is embedded within a superenhancer, ranking 59th of 697 enhancers in their replicate 1 (top 8.5%) and 60th of 638 in replicate 2 (top 9.4%), across a domain of ∼64.7 kb spanning chr9: 57,998,034 to 58,062,777 (mm10) (Fig. 3F). The Cyp11a1 locus exhibits Gata2-dependent open chromatin in resting mast cells and shows further increases in chromatin accessibility and H3K27ac modifications following IgE receptor activation (Fig. 3, E and G). Notably, *Cyp11a1* ranked alongside the canonical mast cell identity genes *Kit*, *Cpa3*, *Gata2*, *Fcer1a*, and *Mitf*, all of which reside within the top-ranked superenhancers, placing the *Cyp11a1* locus within the same tier of transcriptional privilege as the core mast cell identity program (Fig. 3H). Together, these findings establish Cyp11a1 as a superenhancer-associated, Gata2-dependent locus positioned alongside canonical mast cell identity genes, supporting steroidogenesis as a core feature of mast cell biology.

### Cyp11a1 shapes mast cell transcription

To characterize the cell-intrinsic transcriptional consequences of *Cyp11a1* deletion, we performed RNA-seq on control (*Cyp11a1*^fl/fl^) and *Cyp11a1*-deficient (*Cyp11a1*^fl/fl^;*Vav1*^Cre^) BMMCs and PMCs in both mature and postdegranulation recovery phases (Fig. 4A). Principal components analysis revealed distinct transcriptional signatures between control and *Cyp11a1*-deficient cells, with the most pronounced differences emerging during the postdegranulation recovery phase (Fig. 4B). Differential gene expression analysis uncovered widespread transcriptional alterations by *Cyp11a1* deletion, with 321 differentially expressed genes in BMMCs (199 up-regulated and 122 down-regulated) and 664 in PMCs (221 up-regulated and 443 down-regulated) (Fig. 4, C and D, and data S1 and S2). Gene Ontology (GO) enrichment analysis revealed that Cyp11a1-regulated genes were primarily associated with cell division, cell cycle control, and immune response processes in both BMMC and PMC populations (Fig. 4, E and F). In PMCs, the impact was broader, with additional enrichment in pathways related to nuclear division, leukocyte cell-cell adhesion, cytokine-mediated signaling, and T cell activation regulation, indicating wider effects on immune regulatory functions of matured mast cells. Consistent with these transcriptional changes, Cyp11a1-deficient BMMC and PMC cultures yielded significantly fewer cells than controls when equal numbers were initially plated (Fig. 4G). This is consistent with the GO-identified enrichment of cell-cycle programs and indicates that Cyp11a1-dependent steroidogenesis contributes to normal mast cell proliferative capacity and survival during in vitro differentiation.

**Fig. 4.**
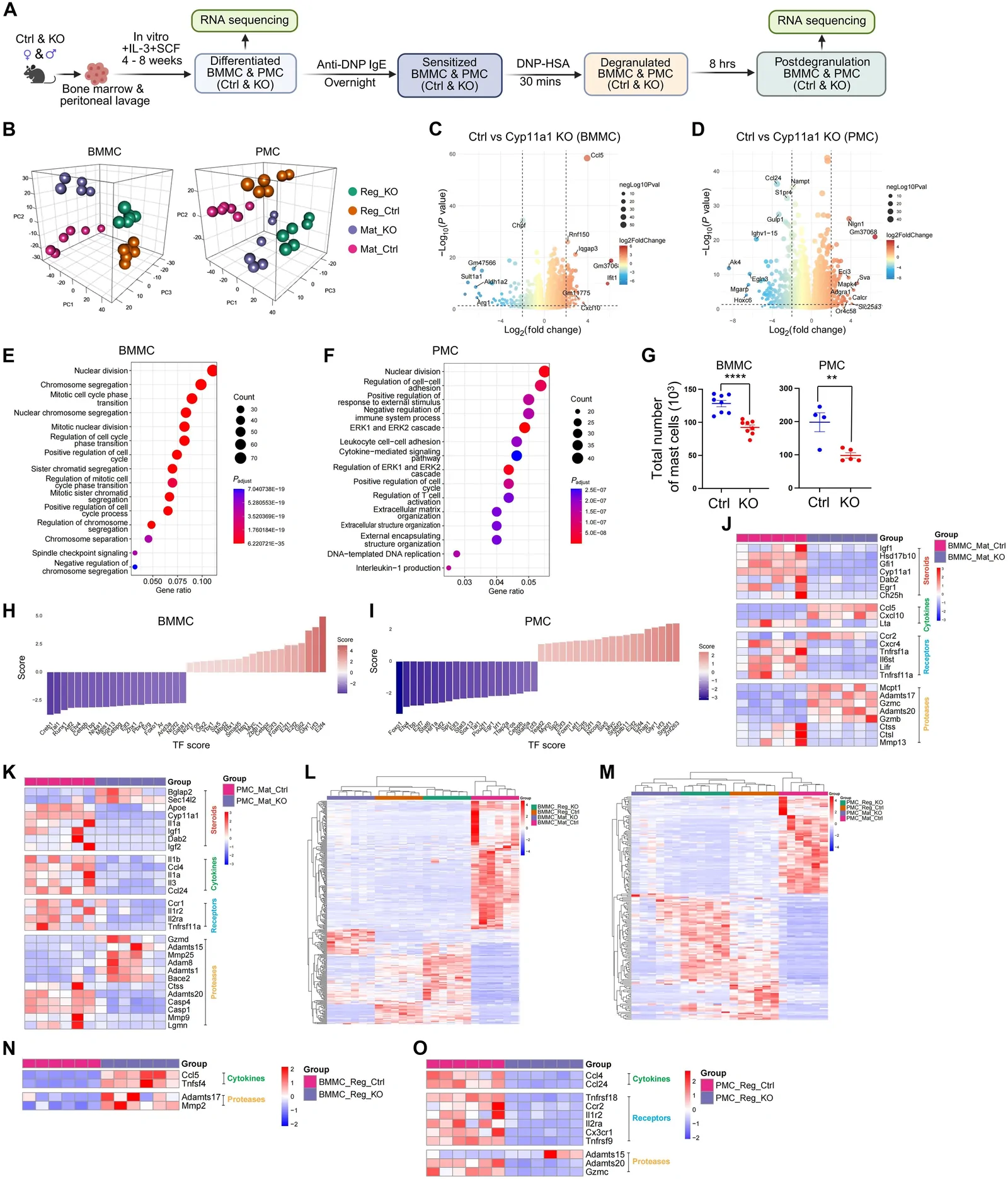
Genome-wide transcriptional effects of Cyp11a1 deletion during mast cell differentiation and postdegranulation recovery. (**A**) Schematic representation of the experimental plan. *Cyp11a1*^fl/fl^ (Ctrl) or *Cyp11a1* ^fl/fl^ *Vav1*^Cre^ (KO) mouse–derived BMMCs and PMCs were in vitro–differentiated and activated. RNA sequencing of mature and 8-hour postdegranulation [regranulating (Reg)] cells was performed and further analyzed bioinformatically. (**B**) Principal components analysis of the transcriptomes. Created in BioRender. B. Mahata (2026), https://BioRender.com/t40u919. (**C** and **D**) Volcano plot depicting differentially expressed genes in control and *Cyp11a1* KO BMMCs (C) and PMCs (D). (**E** and **F**) Gene set enrichment (GO) analysis shows the biological processes in BMMCs (E) and PMCs (F) affected by the *Cyp11a1* KO. (**G**) An equal number of mast cells were plated for BMMC or PMC differentiation. *Cyp11a1* KO mast cell numbers were reduced compared to control mast cells. Representative of four independent experiments. The *P* value was calculated using an unpaired two-tailed *t* test (****P ≤ 0.0001, **P ≤ 0.01). (**H** and **I**) Bar graph representation of the differentially regulated transcription factors (TFs) in BMMCs (H) and PMCs (I) because of *Cyp11a1* deletion. (**J** and **K**) Heatmap representation of the differentially regulated steroid pathway–associated genes, cytokines, receptors, and proteases in BMMCs (J) and PMCs (K) because of *Cyp11a1* deletion. (**L** and **M**) Heatmap showing differentially regulated “postdegranulation-associated regulatory genes” in BMMCs (L) and PMCs (M) following *Cyp11a1* deletion. Genes differentially expressed during the postdegranulation phase, as identified in data S3 and S4. The heatmap visualizes the impact of *Cyp11a1* KO on the expression of these genes. (**N**) Heatmap representation of the differentially regulated cytokines and proteases among the differentially regulated “postdegranulation-associated regulatory genes” in BMMCs [as revealed in (L)] because of *Cyp11a1* deletion. (**O**) Graphical representation of the differentially regulated cytokines, receptors, and proteases among the differentially regulated “postdegranulation-associated regulatory genes” in PMCs [as revealed in (M)] because of *Cyp11a1* deletion.

*Cyp11a1* deletion triggered distinct alterations in transcription factor networks between BMMCs and PMCs (Fig. 4, H and I), with differential regulation of developmental regulators including *Creb1*, *Tal1*, and *Runx1* in BMMCs and *Foxo1*, *Etv4*, and *Tbp* in PMCs. Notably, *Irf3*, a factor known to enhance mast cell degranulation and histamine release during IgE-mediated allergic reactions, was up-regulated in both cell types. These changes were accompanied by cell type–specific alterations in steroid signaling components, cytokines, receptors, and proteases (Fig. 4, J and K). In BMMCs, Cyp11a1 deficiency led to down-regulation of steroid signaling–associated genes (*Igf1*, *Hsd17b10*, and *Gfi1*) while differentially affecting inflammatory mediators and proteases. PMCs exhibited a distinct response pattern, characterized by consistent down-regulation of cytokines (*Il1b*, *Ccl4*, and *Il3*) and cell type–specific alterations in protease expression.

We next compared the transcriptomes of in vitro–generated mast cells (BMMCs and PMCs) before and 8 hours after degranulation and identified differentially expressed genes (data S3 and S4). We refer to these as postdegranulation recovery phase–associated genes. Analysis of this gene set revealed that *Cyp11a1* deletion substantially altered expression patterns in both BMMCs and PMCs (Fig. 4, L and M, and data S5 and S6). *Cyp11a1* deficiency preemptively altered the expression of genes that are normally differentially regulated during the postactivation phase (Fig. 4, L and M). This pattern was observed for key inflammatory mediators including *Ccl5* and *Tnfsf4*, as well as the proteases *Adamts17* and *Mmp2* in BMMCs (Fig. 4N), and cytokines (*Ccl4* and *Ccl24*), receptors (*Il1r2*, *Il2ra*, *Tnfrsf18/19*, and *Cx3cr1*), and proteases (*Adamts15/20* and *Gzmc*) in PMCs (Fig. 4O). These data suggest that Cyp11a1-dependent steroidogenesis contributes to the coordination of transcriptional programs during mast cell differentiation and postactivation recovery.

### Cyp11a1 reshapes the proteomic landscape of mast cells

To assess the proteomic consequences of *Cyp11a1* deletion, we performed quantitative proteomics on control and *Cyp11a1* KO mast cells (BMMCs and PMCs) before and after degranulation (Fig. 5A). This analysis revealed substantial alterations in protein expression resulting from *Cyp11a1* deletion in both mature and postdegranulation states, with distinct changes observed between male and female samples. In mature female BMMCs, *Cyp11a1* deletion resulted in 178 differentially expressed proteins (80 up-regulated and 98 down-regulated) (Fig. 5B and data S7). Male BMMCs showed more modest changes with 30 differentially expressed proteins (Fig. 5C and data S8). PMCs exhibited more extensive alterations, with 329 and 136 differentially expressed proteins in female and male samples, respectively (Fig. 5, D and E, and data S9 and S10).

**Fig. 5.**
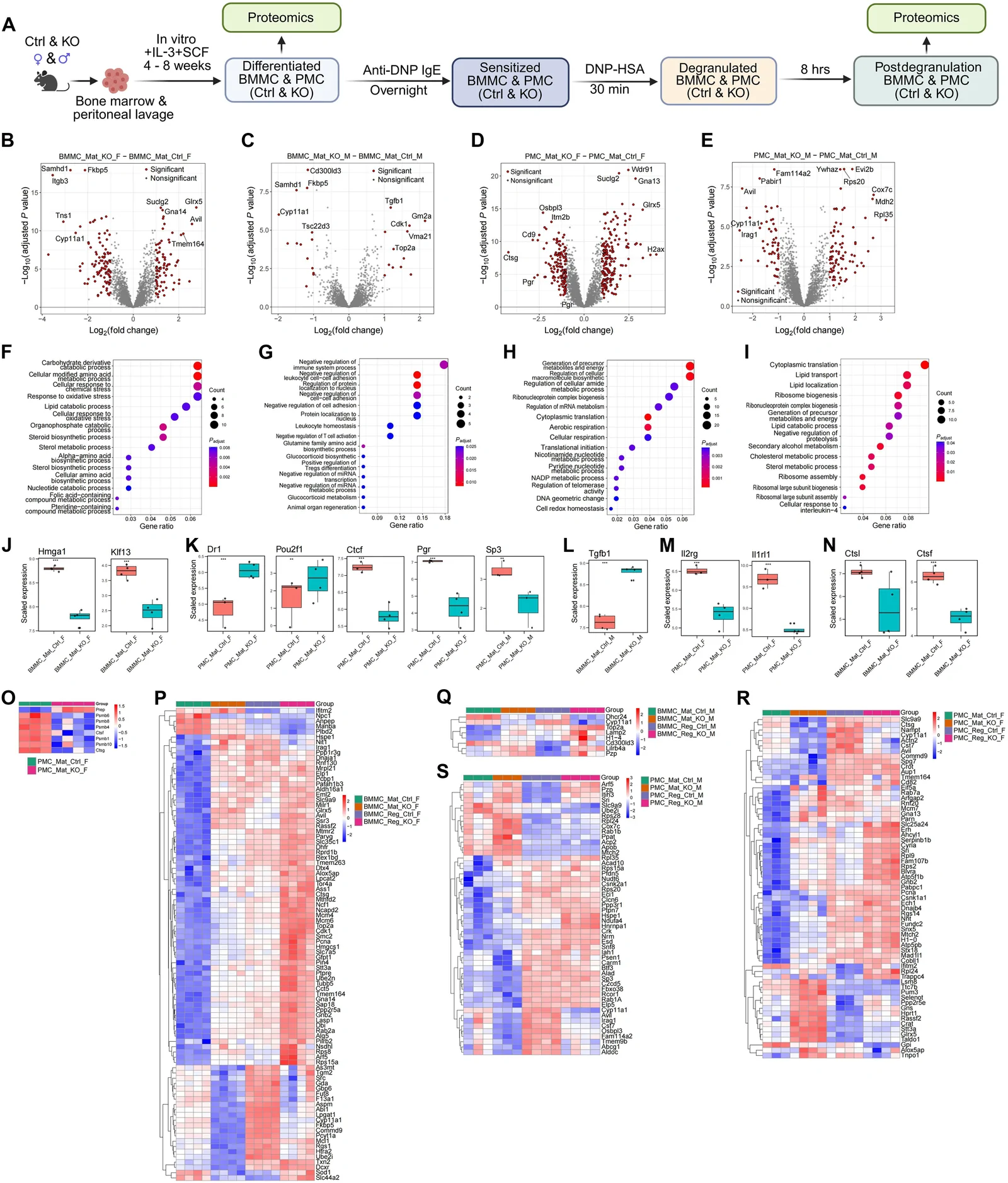
Cyp11a1-dependent steroidogenesis reshapes the proteomic landscape of mast cells. (**A**) Schematic representation of the experimental plan. Control (*Cyp11a1*^fl/fl^) or *Cyp11a1* KO (*Cyp11a1* ^fl/fl^ *Vav1*^Cre^, KO) BMMCs and PMCs were in vitro–differentiated to obtain mature mast cells. Mature mast cells were sensitized by an anti-DNP IgE antibody and activated by exposing them to DNP-HSA. Mature mast cells or mast cells 8 hours after degranulation (BMMCs and PMCs) were used for LC-MS/MS–based proteomics. Created in BioRender. B. Mahata (2026), https://BioRender.com/t40u919. (**B** to **E**) Volcano plots show proteome-wide differentially expressed proteins due to *Cyp11a1* deletion in mature female BMMCs (B), male BMMCs (C), female PMCs (D), and male PMCs (E). (**F** to **I**) GO enrichment analysis in differentially expressed genes upon *Cyp11a1* KO in mature female BMMCs (F), male BMMCs (G), female PMCs (H), and male PMCs (I). (**J**) The graphical representation shows transcription factors’ expression affected by *Cyp11a1* KO in female BMMCs. (**K**) The graphical representation shows transcription factors’ expression affected by *Cyp11a1* KO in mature female (first four panels) and male (last panel, Sp3) PMCs. (**L**) The graphical representation shows cytokines’ expression affected by *Cyp11a1* KO in mature male BMMCs. (**M**) The graphical representation shows cytokine receptor expression affected by *Cyp11a1* KO in mature female PMCs. (**N**) The graphical representation shows proteases’ expression affected by *Cyp11a1* KO in mature female BMMCs. (**O**) The heatmap shows proteases’ expression affected by *Cyp11a1* KO in mature female PMCs. (**P** to **S**) Heatmaps showing differentially expressed proteins upon *Cyp11a1* KO. (P) Female mature versus postdegranulation BMMCs. (Q) Male mature versus postdegranulation BMMCs. (R) Female mature versus postdegranulation PMCs upon *Cyp11a1* KO. (S) Male mature versus postdegranulation PMCs.

GO enrichment analysis of the differentially expressed proteins revealed that Cyp11a1 activity primarily influenced cellular metabolism and immune response pathways in BMMCs, while in PMCs, cellular metabolism– and protein synthesis–associated factors were affected (Fig. 5, F to I). These findings indicate that loss of Cyp11a1-dependent steroidogenesis alters broad cellular programs in mast cells, with a more extensive impact in PMCs than in BMMCs.

Analysis of specific protein categories revealed that *Cyp11a1* deletion affected the expression of several transcription factors in both BMMCs and PMCs (Fig. 5, J and K). Notably, in female BMMCs, factors such as Klf13 and Hmga1 were significantly down-regulated (Fig. 5J). In female PMCs, down-regulator of transcription (Dr1) and Pou2f1 were up-regulated, while Ctcf and progesterone receptor (Pgr) were down-regulated (Fig. 5K, first four panels). Sp3 was down-regulated in male PMCs (Fig. 5K, last panel). The expression of cytokines and cytokine receptors was also modulated by *Cyp11a1* deletion (Fig. 5, L and M). In BMMCs, Tgfb1 showed significant up-regulation (Fig. 5L), while in PMCs, Il2rg and Il1rl1 were differentially expressed (Fig. 5M). These alterations in cytokine and cytokine-receptor expression profiles suggest broader effects of Cyp11a1 on mast cell–mediated communication programs. We observed changes in the expression of proteases upon *Cyp11a1* deletion (Fig. 5, N and O). In female BMMCs, cathepsins such as Ctsl and Ctsf (Fig. 5N) showed differential expression, while in female PMCs, apart from cathepsin, a broader range of proteases was affected (Fig. 5O).

The impact of *Cyp11a1* deletion on the proteome was particularly pronounced during the postdegranulation (regranulation) phase. In female BMMCs, we observed four distinct patterns of protein expression changes (Fig. 5P): (i) Proteins normally up-regulated during postdegranulation were already elevated in Cyp11a1-deficient cells and further increased during postdegranulation, (ii) proteins that normally increase during postdegranulation were down-regulated by Cyp11a1 deletion and failed to respond appropriately, (iii) proteins normally down-regulated during postdegranulation were already reduced in KO cells and further decreased, and (iv) a small subset of proteins normally down-regulated during postdegranulation were paradoxically up-regulated in Cyp11a1-deficient cells. Similar patterns were observed in male BMMCs (Fig. 5Q) and PMCs of both sexes (Fig. 5, R and S), with male PMCs showing particularly extensive effects. These data indicate that Cyp11a1-dependent steroidogenesis shapes the proteomic landscape of mast cells.

### Cyp11a1 supports tissue-resident mast cell abundance

Given that *Cyp11a1* deletion reduced mast cell numbers during in vitro differentiation and propagation and was associated with broad transcriptional and proteomic changes, we next asked whether mast cell–intrinsic steroidogenesis contributes to mast cell distribution in peripheral tissues. To address this, we used *Cyp11a1*^fl/fl^;*Cpa3*^Cre^ mice. The Cpa3-Cre driver (*30*) targets mast cells and basophils and is widely used for efficient deletion in tissue-resident mast cells. We confirmed efficient deletion of Cyp11a1 in mast cells (fig. S4, A and B).

We then quantified mast cells across multiple tissues by flow cytometry and histology. Flow cytometric analysis showed reduced frequencies of mast cells in the peritoneal lavage and small intestine/cecum of KO mice, with a similar downward trend in the lung, whereas no statistically significant reduction was detected in the ear (Fig. 6A). Consistent with this, toluidine blue staining demonstrated significantly reduced mast cell numbers in the tongue and cecum, with a trend toward reduction in ear skin (Fig. 6B). Together, these findings indicate that Cyp11a1-dependent steroidogenesis supports the normal accumulation of mast cells within peripheral tissues.

**Fig. 6.**
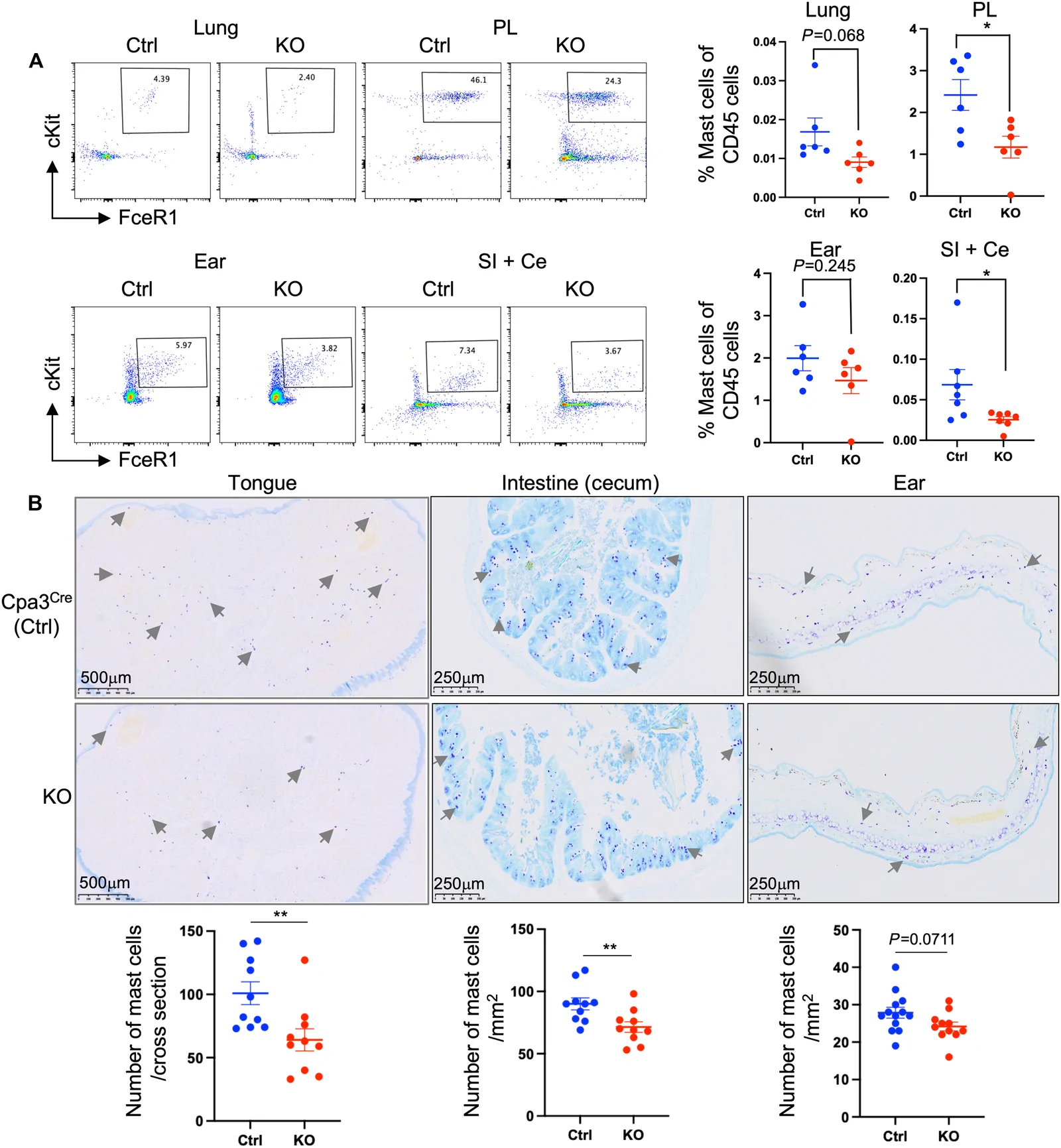
Deletion of *Cyp11a1* reduces the abundance of tissue-resident mast cells. (**A**) Representative flow cytometry plots of FceRI^+^cKit^+^ mast cells in the lung, peritoneal lavage (PL), ear, and small intestine and cecum (SI + Ce) from control (*Cpa3*^Cre^) and KO (*Cyp11a1*^fl/fl^;*Cpa3*^Cre^) mice. Mast cells were quantified as a percentage of CD45^+^ cells. Each dot represents one mouse; bars show the means ± SEM. (**B**) Representative toluidine blue–stained sections of tongue, intestine (cecum), and ear skin from control and KO (*Cyp11a1*^fl/fl^;*Cpa3*^Cre^) mice, with corresponding quantification of mast cell numbers. Mast cells are indicated in the images as marked. Quantification is shown as mast cells per cross section for the tongue and mast cells per square millimeter for the intestine and ear. Scale bars, 500 μm (tongue) and 250 μm (intestine and ear). Each dot represents one mouse; bars show the means ± SEM. Statistical significance is indicated on the graphs. *P* values were calculated using an unpaired two-tailed *t* test (**P ≤ 0.01, *P ≤ 0.05, and exact values for *P* > 0.05).

### De novo steroidogenesis restrains mast cell degranulation

To determine the functional importance of mast cell steroidogenesis, we assessed degranulation responses in Cyp11a1-sufficient and Cyp11a1-deficient mast cells (Fig. 7A). In vitro BMMCs and PMCs were derived from control (*Cyp11a1*^fl/fl^ or *Cpa3*^Cre^ ctrl) and Cyp11a1-deficient (*Cyp11a1*^fl/fl^;*Vav1*^Cre^ or *Cyp11a1*^fl/fl^;*Cpa3*^Cre^) mice, sensitized with anti–dinitrophenyl (DNP) IgE, and activated with DNP-human serum albumin (HSA) (Fig. 7A). Degranulation was quantified as the percentage of β-hexosaminidase released into the supernatant relative to total cellular enzyme activity. Cyp11a1-deficient BMMCs and PMCs exhibited significantly increased degranulation compared to controls in response to IgE-dependent stimulation (Fig. 7B). A similar enhancement of degranulation was observed when cells were activated by the IgE-independent secretagogue compound 48/80, a well-established agonist of the mast cell receptor Mrgprb2 (Fig. 7C). These results indicate that the loss of Cyp11a1-dependent steroidogenesis leads to heightened mast cell degranulation irrespective of the activation pathway. In mature PMCs, basal β-hexosaminidase activity was increased in the cell pellet in the absence of antigen stimulation but not in the supernatant (Fig. 7, D and E). Pharmacological inhibition of Cyp11a1 with aminoglutethimide similarly enhanced β-hexosaminidase release in control cells but not in Cyp11a1-deficient cells, supporting an on-target effect (Fig. 7F). Exogenous pregnenolone suppressed β-hexosaminidase release in both *Cyp11a1*-sufficient and *Cyp11a1*-deficient mast cells and restored *Cyp11a1*-deficient cells toward control levels (Fig. 7G).

**Fig. 7.**
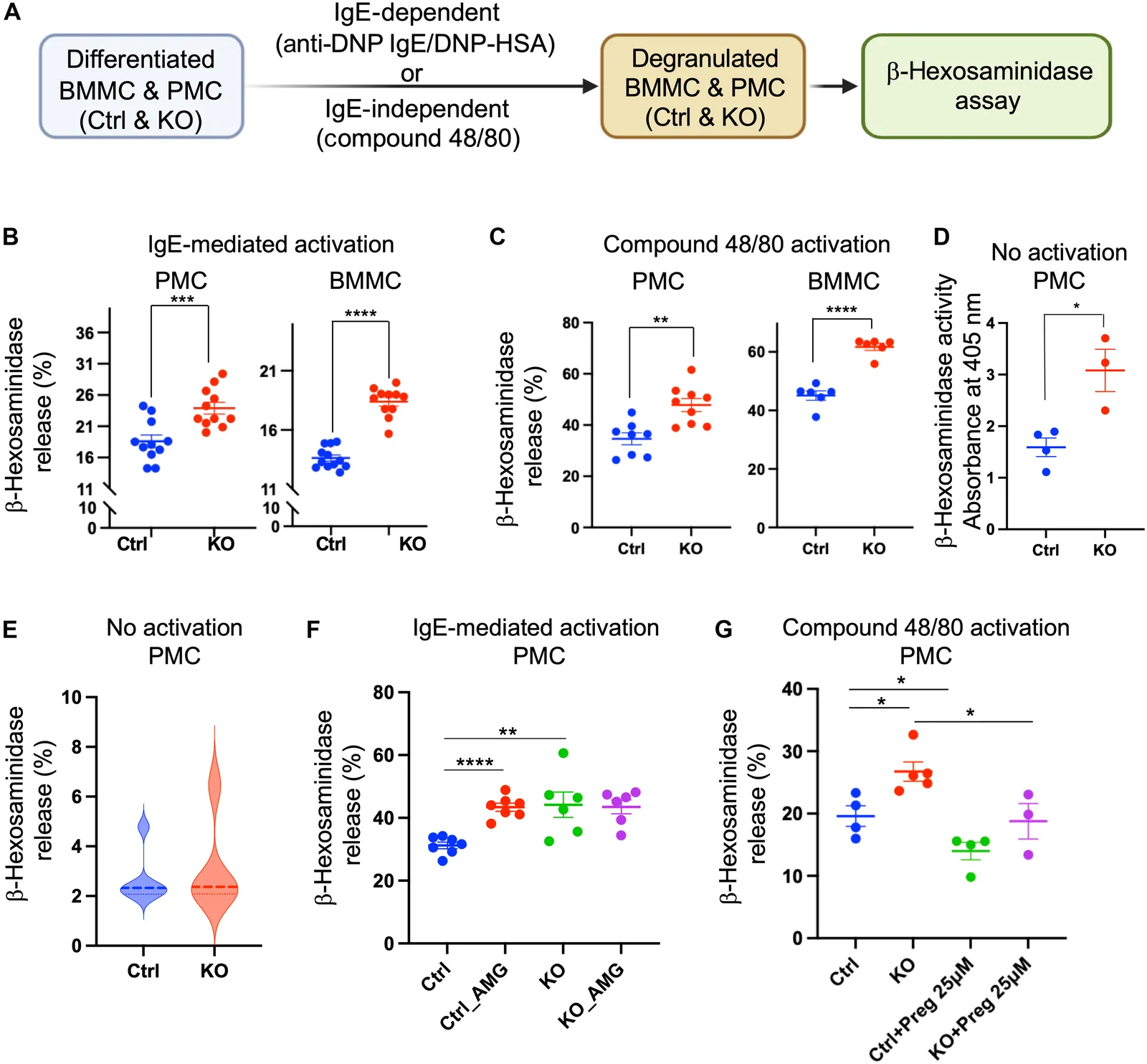
Endogenous steroidogenesis restrains mast cell degranulation. (**A**) Schematic of the experimental design used to assess mast cell degranulation in vitro. Differentiated mast cells from control and Cyp11a1-deficient mice were stimulated through IgE-dependent activation (anti-DNP IgE/DNP-HSA) or IgE-independent activation (compound 48/80), and degranulation was quantified by β-hexosaminidase release assay. Control cells were *Cyp11a1*^fl/fl^ in (B) and *Cpa3*^Cre^ in (C) to (G); Cyp11a1-deficient cells were *Cyp11a1*^fl/fl^;*Vav1*^Cre^ in (B) and *Cyp11a1*^fl/fl^;*Cpa3*^Cre^ in (C) to (G). Created in BioRender. B. Mahata (2026), https://BioRender.com/0nxkxsk. (**B**) IgE-mediated degranulation is increased in Cyp11a1-deficient PMCs (left) and BMMCs (right) compared with control cells, as measured by β-hexosaminidase release. (**C**) IgE-independent degranulation induced by compound 48/80 is increased in Cyp11a1-deficient PMCs (left) and BMMCs (right) compared with control cells. (**D**) Basal granule content is increased in *Cyp11a1*-deficient PMCs compared with control PMCs, as determined by β-hexosaminidase activity in unstimulated cell pellets measured by absorbance at 405 nm. (**E**) Basal spontaneous β-hexosaminidase release in the absence of stimulation is low and not significantly different between control and *Cyp11a1*-deficient PMCs. (**F**) Pharmacological inhibition of Cyp11a1 with aminoglutethimide (AMG; 100 μM) enhances IgE-mediated degranulation in PMCs, phenocopying the effect of genetic *Cyp11a1* deficiency. (**G**) Pregnenolone (25 μM) suppresses compound 48/80–induced degranulation in PMCs and reduces the enhanced degranulation observed in *Cyp11a1*-deficient cells. Representative data are shown from three independent experiments. Statistical significance was determined using unpaired two-tailed Student’s *t* tests (****P ≤ 0.0001, ***P ≤ 0.001, **P ≤ 0.01, *P ≤ 0.05, and ns for *P* > 0.05.

### Cyp11a1 loss exacerbates anaphylactic inflammation in vivo

To validate the above in vitro observations of enhanced degranulation in *Cyp11a1* KO mast cells, in vivo, we performed passive cutaneous anaphylaxis (PCA) reactions in *Cyp11a1*^fl/fl^;*Cpa3*^Cre^ (KO) mice and *Cpa3*^Cre^ controls. *Cpa3* is also expressed in basophils; however, the PCA reaction is a well-established model of mast cell–dependent inflammation.

In the PCA model, ear skin mast cells were locally sensitized by intradermal injection of anti-DNP IgE, and cutaneous anaphylaxis was induced 24 hours later by intravenous challenge with DNP-HSA together with Evans blue dye (Fig. 8A). *Cyp11a1*-deficient mice displayed markedly enhanced inflammatory responses characterized by significantly increased ear swelling (Fig. 8B), elevated vascular permeability as quantified by Evans blue extravasation (Fig. 8C), and increased mast cell degranulation revealed by toluidine blue histopathology (Fig. 8, D and E, and fig. S5). These in vivo results demonstrate that mast cell–intrinsic Cyp11a1-dependent steroidogenesis functions as an endogenous negative regulatory mechanism that limits the magnitude of anaphylactic inflammatory responses.

**Fig. 8.**
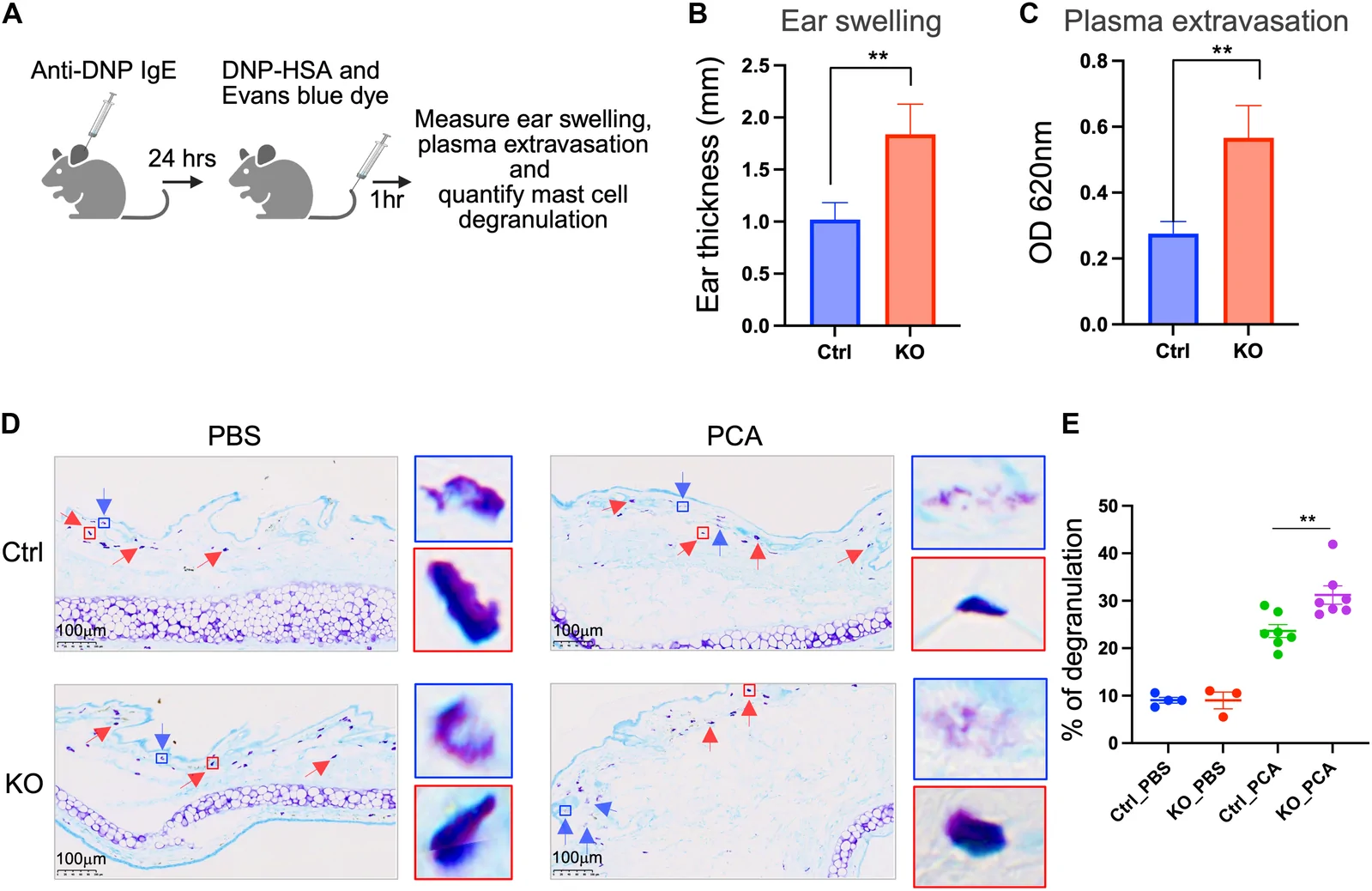
Cyp11a1 deletion in Cpa3-expressing cells exacerbates passive cutaneous anaphylaxis. (**A**) Schematic of the passive cutaneous anaphylaxis (PCA) protocol. Ears of control (*Cpa3*^Cre^) and Cyp11a1-deficient (*Cyp11a1*^fl/fl^;*Cpa3*^Cre^) mice were sensitized by intradermal anti-DNP IgE injection. After 24 hours, mice were challenged intravenously with DNP-HSA together with Evans blue dye, and ear swelling and plasma extravasation were assessed 1 hour later. Created in BioRender. B. Mahata (2026), https://BioRender.com/t40u919. (**B**) Ear swelling was increased in Cyp11a1-deficient mice compared with control mice following PCA challenge, as measured by ear thickness. (**C**) Vascular permeability was increased in *Cyp11a1*-deficient mice compared with control mice following PCA challenge, as determined by Evans blue extravasation quantified by absorbance at 620 nm. OD, optical density. (**D**) Representative toluidine blue–stained ear sections from PBS- and PCA-treated control and *Cyp11a1*-deficient mice. Degranulating mast cells (blue boxes and arrows) are characterized by loss of cytoplasmic granule density with pale, diffuse staining. Intact, nondegranulated mast cells (red boxes and arrows) are characterized by dense metachromatic (purple-red) granular staining on toluidine blue. Scale bars, 100 μm. (**E**) Quantification of mast cell degranulation in toluidine blue–stained ear sections, expressed as the percentage of degranulated mast cells. PCA induced mast cell degranulation in both genotypes, with a greater response in *Cyp11a1*-deficient mice than in controls. Data are representative of three independent experiments. Statistical significance was determined using an unpaired two-tailed Student’s *t* test (**P ≤ 0.01 and ns for *P* > 0.05).

## DISCUSSION

This study reveals a previously unrecognized cell-intrinsic regulatory pathway in mast cells: the constitutive production of pregnenolone through Cyp11a1-dependent steroidogenesis. This pathway is embedded in the mast cell identity program through Gata2 and superenhancer regulation, reshapes transcriptional and proteomic programs during activation, and restrains degranulation and anaphylactic inflammation in vivo. Collectively, these findings reposition mast cells as both targets and local producers of steroid signals and reveal an endogenous feedback mechanism that limits their inflammatory output.

### Cell-intrinsic steroidogenesis as a negative regulator of mast cell function

Our functional data demonstrate that mast cell–intrinsic steroidogenesis acts as a brake on degranulation. Cyp11a1-deficient mast cells responded excessively to both IgE-dependent and IgE-independent stimuli, and this hyperresponsiveness was confirmed in vivo by enhanced passive cutaneous anaphylaxis. The convergence of genetic loss, pharmacological inhibition, and pregnenolone rescue provides strong evidence for a model in which endogenous pregnenolone production dampens mast cell effector function. Notably, this increased per-cell reactivity was sufficient to enhance anaphylactic severity despite reduced mast cell abundance in several tissues, indicating that steroidogenesis restrains the amplitude of inflammatory responses at the level of individual cells.

### Gata2-mediated transcriptional regulation of mast cell steroidogenesis

Our regulatory analyses place *Cyp11a1* within the core transcriptional architecture of mast cells. ChIP-seq and ATAC-seq (assay for transposase-accessible chromatin with high-throughput sequencing) data demonstrate that Gata2 binds at multiple sites across the Cyp11a1 locus, upstream of the transcription start site, at promoter-proximal regions, and within intronic regions, and that chromatin accessibility at this locus is abolished in Gata2-deficient BMMCs (*25*). Furthermore, the Cyp11a1 locus is embedded within a superenhancer that ranks among the top 9% of all enhancers in resting BMMCs, alongside canonical identity genes including *Kit*, *Cpa3*, *Gata2*, *Fcer1a*, and *Mitf*. This positioning argues that steroidogenesis is transcriptionally privileged to the same degree as core mast cell effector programs. *Cyp11a1* up-regulation during the postdegranulation recovery phase further suggests that Gata2-driven steroidogenesis is dynamically regulated across the mast cell activation cycle, potentially providing a mechanism by which mast cells modulate their own responsiveness to subsequent activation.

### Insights into the postdegranulation recovery phase, a critical period for immune homeostasis

Mast cells are long-lived cells with the capacity for multiple rounds of activation and degranulation. Our study provides molecular insights into the postdegranulation recovery phase, a critical period during which these cells reset their transcriptional and proteomic states after activation to restore their secretory capacity and to contribute to the resolution of inflammation and tissue repair. The data show that Cyp11a1 expression and pregnenolone production increase during this phase and that loss of Cyp11a1 alters multiple gene and protein programs associated with postactivation recovery. While these findings support a role for Cyp11a1-dependent steroidogenesis in shaping the postactivation state of mast cells, they do not by themselves establish a direct functional role in regranulation or tissue repair, which will require dedicated future studies.

### Therapeutic implications of mast cell steroidogenesis

The identification of Cyp11a1-dependent steroidogenesis as an endogenous negative regulator of mast cell activation raises the possibility that this pathway could be therapeutically exploited in allergic or inflammatory disease. At present, however, the data support this idea only at the level of biological plausibility. Whether mast cell–intrinsic steroidogenesis can be targeted selectively, and whether this would provide advantages over existing anti-inflammatory therapies, will require further testing in disease models and pharmacological studies.

### Limitations of the study and future direction

Transcriptomic and proteomic analyses were performed on cultured BMMCs and PMCs, which, although well-established and informative, do not fully capture the phenotypic diversity and tissue imprinting of in vivo mast cell populations. The Cpa3-Cre driver used for in vivo experiments also targets basophils, and while the PCA model is well characterized as mast cell–dependent, a contribution from basophil-intrinsic *Cyp11a1* deficiency cannot be entirely excluded. In addition, *Cyp11a1* was deleted before mast cell maturation, leaving open the possibility that some phenotypes reflect altered development as well as loss of acute steroidogenic function. Future studies using inducible deletion in mature mast cells and reconstitution of mast cell–deficient hosts with *Cyp11a1*-deficient cells will be important for resolving these questions.

In conclusion, our study identifies Cyp11a1-dependent pregnenolone biosynthesis as a cell-intrinsic regulatory pathway that is embedded in the mast cell identity program, reshapes molecular programs during activation and recovery, and restrains degranulation and anaphylactic inflammation in vivo. These findings reveal an endogenous steroidogenic feedback mechanism in mast cells and provide a rationale for exploring mast cell–intrinsic steroid signaling as a target for more selective interventions in allergic and inflammatory disease.

## MATERIALS AND METHODS

### Mice

In this investigation, all mice were handled and cared for following the stringent guidelines set forth by the UK Animals in Science Regulation Unit’s Code of Practice for the Housing and Care of Animals Bred, Supplied, or Used for Scientific Purposes, and the Animals (Scientific Procedures) Act 1986 Amendment Regulations 2012. All experimental protocols were conducted under the authorization of a UK Home Office Project License (PPL PP2448972 and PP4938782) and received the necessary approval from the local institute’s Animal Welfare and Ethical Review Body, ensuring compliance with ethical standards and animal welfare considerations. The sample size was determined according to our previous experience and a priori power analysis (G*Power). Regarding the housing condition of Gurdon animal facility, all mice used in this study were maintained in a specific pathogen–free unit on 12 hours of light and 12 hours of dark cycles. The mice were genotyped by Transnetyx. *Cyp11a1-*mCherry reporter and *Cyp11a1*^fl/fl^ mice were generated by Sanger Institute and previously reported (*19*). Mast cell– and basophil-specific KO mice, *Cyp11a1*^fl/fl^;*Cpa3*^Cre^, were generated by crossing with *Cpa3-*Cre mice (the Jackson Laboratory). In this study, we used mice aged between 8 and 12 weeks.

### BMMC differentiation and culture

Bone marrow cells were isolated from femurs and tibias of 6- to 12-week-old mice and cultured in RPMI 1640 medium supplemented with 10% fetal bovine serum (FBS), penicillin (100 U/ml), streptomycin (100 μg/ml), 2 mM l-glutamine, 50 μM 2-mercaptoethanol, IL-3 (30 ng/ml), and stem cell factor (SCF; 10 ng/ml). Cells were incubated at 37°C and 5% CO_2_ for 4 to 6 weeks. Media were changed on second and fourth days, and cells were transferred to a new flask followed by regular medium change (every 5 to 7 days). Mast cell purity (>95%) was confirmed by flow cytometry analysis of FcεRI and c-Kit expression.

### PMC isolation and culture

We used 8- to 12-week-old female C57BL/6N mice. After culling, 7 ml of ice-cold sterile Tyrode’s buffer (Thermo Fisher Scientific, cat. no. J67607.K2) and 3 ml of air were injected in the peritoneal cavity using a 10-ml syringe equipped with a 27G needle. After injection, we shook the mice for 1 min to detach peritoneal cells into Tyrode’s buffer. We aspirated the fluid from the abdomen gently and slowly (∼0.5 ml/s) to avoid clogging by the inner organs and transferred the collected cell suspension into a collection tube. Next, we centrifuged the tubes with the cell suspension at ∼300*g* for 7 min at 4°C. Under a sterile hood, we aspirated the supernatant and washed cells with complete Dulbecco’s modified Eagle’s medium. We resuspended the pellets in 4 ml of prewarmed complete Dulbecco’s modified Eagle’s medium with IL-3 (20 ng/ml) and SCF (30 ng/ml), transferred the cell suspension to a 25-cm^2^ culture flask, and incubated for 2 to 4 weeks (37°C and 5% CO_2_). On second and fourth days after plating cells, we changed the medium, transferred the cell suspension on to a new culture flask, and continued the culture for 2 to 4 weeks. We analyzed their maturation (cKit and FceR1 expression) by flow cytometry.

### Mast cell degranulation assay

Mast cell degranulation was assessed by measuring β-hexosaminidase release as previously described (*31*), with modifications. BMMCs and PMCs were sensitized overnight with anti-DNP IgE (1 μg/ml), washed, and resuspended in Tyrode’s buffer at a concentration of 1 × 10^6^ cells/ml. Cells were then stimulated with DNP-HSA (10 to 100 ng/ml). Compound 48/80 (10 μg/ml) was used for IgE-independent degranulation for 30 min in Tyrode’s buffer. After stimulation, cell suspensions were centrifuged and placed on ice, and supernatants were collected. The cell pellets of unstimulated cells were lysed with 0.5% Triton X-100 to determine the maximal enzymatic activity of β-hexosaminidase. Twenty-five microliters of supernatant or lysate volume corresponding to 5 × 10^3^ cells was incubated with 50 μl of a *p*-nitrophenyl-*N*-acetyl-β-d-glucosaminide (also known as pNAG) solution (1.3 mg/ml) in 50 mM citrate buffer (pH 4.5) at 37°C for 90 min. The reaction was stopped with 150 μl of 0.2 M glycine buffer (pH 10.7) and absorbance read at 405 nm. The β-hexosaminidase activity present in the supernatants and in cell pellets was then measured, and the percentage of β-hexosaminidase release (degranulation) was calculated: Percentage degranulation = 100 × (supernatant activity)/(supernatant activity + lysate activity) (*24*).

### Spectral flow cytometry

We followed eBioscience surface staining and intracellular cytoplasmic protein staining (for cytokines) protocols. Briefly, single-cell suspensions were stained with the Live/Dead Fixable Dead cell stain kit (Molecular Probes/Thermo Fisher Scientific) and blocked by purified rat anti-mouse CD16/CD32 purchased from BD Bioscience and eBioscience. Surface staining was performed in flow cytometry staining buffer (eBioscience) or in phosphate-buffered saline (PBS) containing 3% FBS at 4°C. After staining, cells were washed with flow cytometry staining buffer (eBioscience) or 3% PBS-FBS and were analyzed by Cytek Aurora (5L) flow cytometer. FlowJo version 10.2 software facilitated the data analysis.

### Steroid quantification via LC-MS (LC-MS/MS)

Samples (100 μl) of each cell supernatant sample were enriched with isotopically labeled internal standards, including ^13^C_2_,d_2_-pregnenolone (13C2,d2-P5; 1 ng), and extracted along with a mixed steroid calibration curve, including pregnenolone (0.005 to 1 ng), through supported liquid extraction plates on an Extrahera liquid handling robot (Biotage, Uppsala, Sweden) using dichloromethane/isopropanol (98:2, v/v), reduced to dryness under nitrogen and resuspension in water/methanol [80 μl; 70:30 (v/v) water/methanol] followed by LC-MS/MS analysis of the extract.

Frozen tissue (∼50 mg—exact weight recorded) was placed in 2-ml reinforced tubes (containing 1.4-mm ceramic beads, Thermo Fisher Scientific). Acetonitrile with 0.1% formic acid (v/v; 1 ml) was added to the tube, and it was enriched with 20 μl of isotopically labeled steroid standard mixture (as above). Each tube was added to a Bead Ruptor 24 Elite (Omni International) fitted with a CryoCool unit. The tubes were homogenized for 1 m/s for 30 s for three cycles. The supernatant for each sample was transferred to a Filter+ plate (Biotage, Sweden), positive pressure was applied, and the eluate was collected in a clean 96-well collection plate. The filtered homogenate was further processed through a phospholipid depletion (PLD+) plate (Biotage, Sweden), and the eluate was reduced to dryness and resuspended in water/methanol (70:30, v/v), and the plate was sealed with a zone-free plate seal, ready for LC-MS/MS analysis.

Briefly, an I-Class UPLC (Waters, UK) was used for the liquid chromatography on a Kinetex C18 column (150 by 2.1 mm; 2.6 μm) with a flow rate of 0.3 ml/min and a mobile phase system of water with 0.05 mM ammonium fluoride and methanol with 0.05 mM ammonium fluoride, starting at 50% B, rising to 95% B, and returning to 50% B. Separation of 18 steroids was carried out. The column and autosampler temperatures were maintained at 50° and 10°C, respectively. The injection volume was 20 μl, and the total analytical run time per sample was 16 min. Steroids were detected on a QTrap 6500+ mass spectrometer (AB Sciex, Warrington, UK) equipped with an electrospray ionization turbo V ion spray source. The positive ion spray voltage was set to 5500 V, and the negative ion spray voltage was set to −4500 V, with the source temperature maintained at 600°C. Multiple reaction monitoring parameters were carried out for all steroids including pregnenolone (P5) at mass/charge ratio (*m*/*z*) values of 317.1, 281.1, and 159.0 with a declustering potential of 66, collision exit potential values of 31 and 29 V, and a collision energy of 12 V and for 13C2,d2-P5 of 321.2 × 285.2 with a declustering potential of 14, a collision exit potential of 17, and a collision energy of 18 with a retention time of 10.4 min.

The ratio of P5/13C2,d2-P5 peak areas were calculated, and linear regression analysis was used to calculate the amount of P5 in each sample. The same was done for other steroids in the sample (aldosterone, progesterone, 17β-estradiol, 5α-dihydrotestosterone, and testosterone) by evaluation of the data on MultiQuant 3.0.3 (AB Sciex, UK).

### Oxysterol quantification via LC-MS (LC-MS/MS)

The method for sterol extraction, derivatization, and LC-MS has been previously described in detail (*32*). Briefly, oxysterols in cell pellets were extracted in ethanol containing [26,26,26,27,27,27-^2^H_6_]24*R*/*S*-hydroxycholesterol ([2H6]24R/S-HC, Avanti Polar Lipids). Oxysterols were separated from cholesterol by C18 solid-phase extraction and derivatized with Girard P reagent (Tokyo Chemical Industry) with or without prior oxidation of 3-hydroxy to 3-oxo groups. The oxysterols were analyzed using LC-MS with multistage fragmentation [LC-MS(MSn)] on an Orbitrap IDX mass spectrometer coupled with THE Ultimate 3000 LC system (Thermo Fisher Scientific). The identification of 22R-HC and 20R,22R-diHC was based on exact mass, retention time, and comparison of MS3 fragmentation spectra to authentic standards (22R-HC, Avanti Polar Lipids, 20R,22R-diHC, a gift from I. Björkhem, Karolinska Institute). The two oxysterols were quantified against the internal standard [2H6]24R/S-HC.

### Bulk RNA-seq analysis

Quality assessment of the raw sequencing reads was conducted using FastQC, and the HISAT2 aligner was used to map the reads to the mouse genome GRCm38 (mm10), resulting in SAM files for each sample. Samtools was subsequently used to convert and sort these files into BAM format. Gene-level abundance and raw counts were derived using htseq-count, providing the necessary data for principal components analysis and differential gene expression analysis in DESeq2 (version 1.30.1) (*33*). Gene set enrichment analysis (*34*) and gene pathway enrichment were performed with clusterProfiler (version 3.18.1) (*35*). Transcription factor analysis was performed using decoupleR (version 2.4.0) (*36*, *37*).

The RNA-seq dataset of human skin mast cells was obtained from the Gene Expression Omnibus (GEO) under accession number GSE217060 (*38*). Normalized count data provided by the authors were used to visualize selected gene expression.

### Single-cell RNA-seq reanalysis

The single-cell RNA-seq datasets of pan-organ murine mast cells were obtained from the nonprofit repository Dryad (https://doi.org/10.5061/dryad.np5hqbzzz) (*20*). Gene expression visualization was performed using Seurat version 3.2.2. (*39*).

### ChIP seq and omni-ATAC seq data analysis

ChIP-seq data for Gata2 binding in primary mast cells were obtained from the GEO under accession number GSE48086 (*26*). The corresponding BED file indicating Gata2-bound regions was visualized using the UCSC Genome Browser, aligned to the *Mus musculus* mm9 reference genome.

ATAC-seq data were retrieved from GEO accession number GSE145612 (*25*). Processed coverage data in .tdf format were extracted from .bdg.tdf files and visualized using the Integrative Genomics Viewer (IGV), with alignment to the *M. musculus* mm10 reference genome. For each sample, read coverage was displayed on a normalized scale ranging from 0 to 50.

### Passive cutaneous anaphylaxis (PCA) reaction in mice ear

Six- to eight-week-old *Cpa3*^Cre^ (control) or Cyp11a1 KO mice (*Cyp11a1*^fl/fl^;*Cpa3*^Cre^) were sensitized by intradermal injection of 0.4 μg of anti-DNP IgE in 10 μl of PBS or PBS only into the ear. After 24 hours, mice were challenged intravenously via the tail vein with 200 μl of a solution containing 10 μg of DNP-HSA and 2% Evans blue dye in PBS. Ear thickness was measured using a digital caliper immediately before and 1 hour after the challenge. One hour postchallenge, mice were euthanized, and ears were excised. Each ear was homogenized in 1 ml of PBS, followed by the addition of acetone (3:7, v/v). After overnight incubation at room temperature, samples were centrifuged at 3000 rpm for 15 min. The absorbance of the supernatant was measured at 620 nm to quantify Evans blue as a surrogate of plasma extravasation.

### Toluidine blue staining

Tissue samples from mouse ears, tongue, and cecum were fixed in 10% neutral-buffered formalin for 24 hours at room temperature. Fixed tissues were dehydrated through a graded ethanol series, cleared in xylene, and embedded in paraffin. Sections of 4 to 8 μm in thickness were cut using a microtome and mounted on glass slides. For toluidine blue staining, sections were deparaffinized in xylene and rehydrated through a graded ethanol series. Slides were then stained with 0.1% Toluidine Blue O solution (pH 2.0 to 2.5) for 5 to 10 min at room temperature. After rinsing in distilled water, sections were rapidly dehydrated through 95 and 100% ethanol, cleared in xylene, and mounted with a coverslip using a permanent mounting medium. This method results in metachromatic staining of mast cell granules, appearing purple-red against a blue background. Mast cells were quantified by counting toluidine blue–positive cells in multiple high-power fields per section using light microscopy. We used a NanoZoomer to capture images, and figures were made using NDP.view2 software.

### Sample dissolution, TMT labeling, and reverse-phase fractionation for proteomics

Tissue samples were resuspended in lysis buffer containing 100 mM triethylammonium bicarbonate (Sigma-Aldrich), 10% isopropanol, 50 mM NaCl, and 1% sodium deoxycholate with Nuclease and Protease/Phosphatase Inhibitors, followed by 15-min incubation at room temperature. The protein concentration was estimated using Bradford assay according to the manufacturer’s instructions (BIO-RAD-Quick start). Total proteins were reduced and alkylated with 2 μl of 50 mM tris-2-caraboxymethyl phosphine (Sigma-Aldrich) and 1 μl of 200 mM iodoacetamide (Sigma-Aldrich) for 1 hour. Then, protein samples were digested overnight at 37°C using trypsin solution at a protein/trypsin ratio of ∼1:30. The next day, protein digest was labeled with the TMTpro reagents (Thermo Fisher Scientific) for 1 hour and quenched with 5% hydroxylamine (Thermo Fisher Scientific) for 15 min at room temperature. All the samples were mixed and acidified using formic acid for sodium deoxycholate removal. The dry TMT mix was fractionated on a Dionex Ultimate 3000 system at high pH using the X-Bridge C18 column (3.5 μm, 2.1 by 150 mm, Waters) with a 90-min linear gradient from 5 to 95% acetonitrile containing 20 mM ammonium hydroxide at a flow rate of 0.2 ml/min. Peptide fractions were collected between 20 and 55 min and were dried with a speed vac concentrator. The fractions were reconstituted in 0.1% formic acid for LC-MS/MS analysis (*40*).

#### LC-MS/MS for proteomics

Peptide fractions were analyzed on a Dionex Ultimate 3000 system coupled with the nano-ESI source Fusion Lumos Orbitrap Mass Spectrometer (Thermo Fisher Scientific). Peptides were trapped on a 100 μm inside diameter–by–2 cm microcapillary C18 column (5 μm, 100A) followed by 2-hour elution using 75 μm inside diameter–by–25 cm C18 RP column (3 μm, 100A) at a flow rate of 300 nl/min. In each data collection cycle, one full MS scan (380 to 1500 *m*/*z*) was acquired in the Orbitrap [120,000 resolution, automatic gain control (AGC) setting of 3 × 10^5^, and maximum injection time (MIT) of 100 ms]. The subsequent MS2 was conducted with a top speed approach using a 3-s duration. The most abundant ions were selected for fragmentation by collision-induced dissociation (CID). CID was performed with a collision energy of 35%, an AGC setting of 1 × 10^4^, an isolation window of 0.7 Da, and a MIT of 35 ms. Previously analyzed precursor ions were dynamically excluded for 45 s. During the MS3 analyses for TMT quantification, precursor ion selection was based on the previous MS2 scan and isolated using a 2.0-Da *m*/*z* window. MS2-MS3 was conducted using sequential precursor selection methodology with the top 10 settings. Higher-energy collisional dissociation was used for the MS3, it was performed using 55% collision energy, and reporter ions were detected using the Orbitrap (50,000 resolution, an AGC setting of 5 × 10^4^, and a MIT of 86 ms). Phospho fractions were subjected to MS2 analysis only.

#### Data processing

Proteome Discoverer 3.0. (Thermo Fisher Scientific) was used for the processing of CID tandem mass spectra. The SequestHT search engine was used, and all the spectra were searched against the UniProt *M. musculus* FASTA database (taxon ID 10090—version June 2023). All searches were performed using a static modification of TMTpro (+304.207 Da) at any N terminus and lysines and carbamidomethyl at cysteines (+57.021 Da). Methionine oxidation (+15.9949 Da), phospho (+79.966 Da on serine, threonine, and tyrosine), and deamidation on asparagine and glutamine (+0.984) were included as dynamic modifications. Mass spectra were searched using the precursor ion tolerance of 20 ppm (parts per million) and fragment ion tolerance of 0.5 Da. For peptide confidence, 1% false discovery rate was applied, and peptides uniquely matched to a protein were used for quantification. Analysis was carried out using the qPLEXanalyzer package (version 1.16.1) in R (*41*).

### Quantitative pregnenolone ELISA

Pregnenolone concentrations of the cell culture supernatants were quantified using a pregnenolone enzyme-linked immunosorbent assay (ELISA) kit (Abnova) following the manufacturers’ instructions. Absorbance was measured at 450 nm, and data were analyzed in GraphPad Prism 6.

### Statistics

For the calculation of differentially expressed genes in bulk RNA-seq datasets, *P* values were derived using the default methodologies embedded in the respective R packages. In both in vitro and in vivo experimental settings, Prism9 (GraphPad Software) was used to ascertain the significance levels of the data, applying a two-tailed Student’s *t* test. A *P* value falling below the 0.05 threshold was deemed to indicate statistical significance.
