# Supplementary material for: Cell-intrinsic steroidogenesis regulates mast cell function
Source: Sci Adv. 2026 Oct 16;12(42):eaea0260. doi: 10.1126/sciadv.aea0260 (PMC13674488; doi:10.1126/sciadv.aea0260)
Supplement: Supplementary file 1 [file sciadv.aea0260_sm.pdf]

Science Advances
AAAS

Supplementary Materials for

Cell-intrinsic steroidogenesis regulates mast cell function

Jhuma Pramanik *et al.*

Corresponding author: Jhuma Pramanik, jp822@cam.ac.uk; Bidesh Mahata, bm562@cam.ac.uk

Sci. Adv. **12**, eaea0260 (2026)
DOI: 10.1126/sciadv.aea0260

The PDF file includes:

Figs. S1 to S5
Legends for data S1 to S10

Other Supplementary Material for this manuscript includes the following:

Data S1 to S10

Fig. S1

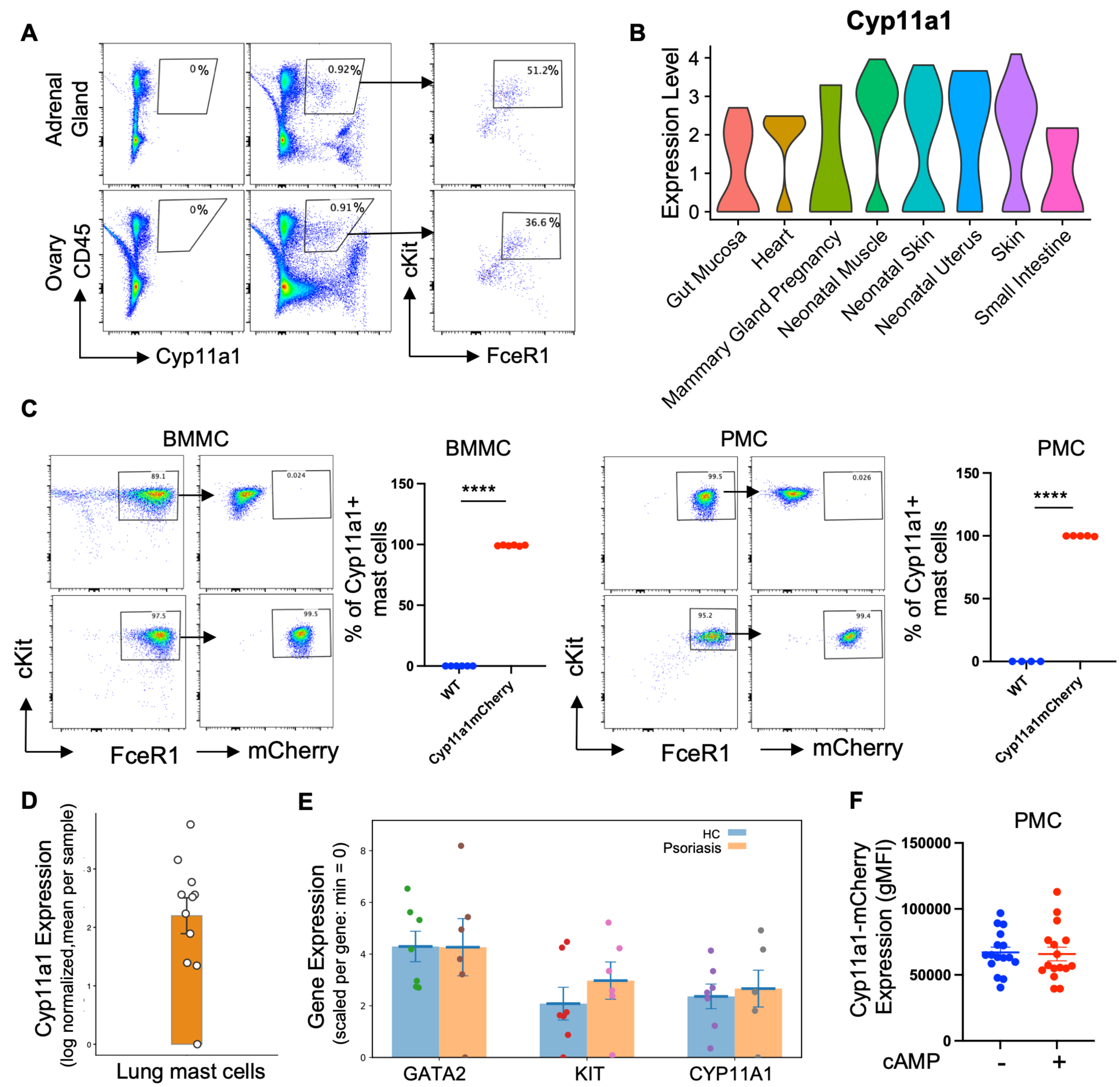

Fig S1. Cyp11a1 expression by mast cells. (A) Representative FACS plots of Cyp11a1-mCherry expression in steroidogenic tissue (adrenal gland and ovary)-resident mast cells. FACS gating: All cells > Singlets > live cells > Lineage$^-$ > CD45+ Cyp11a1 mCherry$^+$ > cKit$^+$FceR1$^+$ **(B)** Violin plots showing *Cyp11a1* expression in mast cells across diverse mouse tissues. Single-cell RNA sequencing data from, gut mucosa, heart, pregnant mammary gland, neonatal muscle, skin, uterus, peritoneal cavity, and small intestine were analysed. Plot width indicates cell density at each expression level. RNAseq data were obtained from public repositories (see Methods). **(C)** Flow cytometric analysis of Cyp11a1 expression in bone marrow-derived (BMMC) and peritoneal (PMC) mast cells. Cells were differentiated *in vitro* from Cyp11a1-mCherry reporter or wild-type (WT) mice. FACS plots (representative) and histograms show

mCherry expression as a surrogate for Cyp11a1. Representative of four independent experiments. p-value was calculated using unpaired two-tailed t-test (**** for $p \leq 0.0001$). **(D)** *Cyp11a1* expression in lung mast cells. Bar plot showing *Cyp11a1* expression in mouse lung mast cells, queried from the LungMAP Single Cell Reference CellRef dataset. Expression is shown as log-normalised mean expression per sample. The orange bar represents the mean expression across mast cell samples, with overlaid open circles indicating individual samples and the error bar indicating SEM. **(E)** Key mast cell identity genes and *CYP11A1* expression in human mast cells. *GATA2*, *KIT* and *CYP11A1* expression in human mast-cell of healthy control (HC) and psoriasis patients' skin samples. Grouped bar plots show scaled expression (± SEM) of *GATA2, KIT* and *CYP11A1* across samples from healthy controls (HC; H1–H7) and psoriasis (L1–L6). Bars denote group means; error bars indicate SEM; thick horizontal lines mark the mean for each group. **(F)** Effect of cAMP (8-Br-cAMP, 500µM) on Cyp11a1 expression in peritoneal mast cells. Cyp11a1-mcherry mice derived PMCs were cultured with or without cAMP for 16 hours and Cyp11a1-mCherry expression was detected by flow cytometry.

Fig. S2

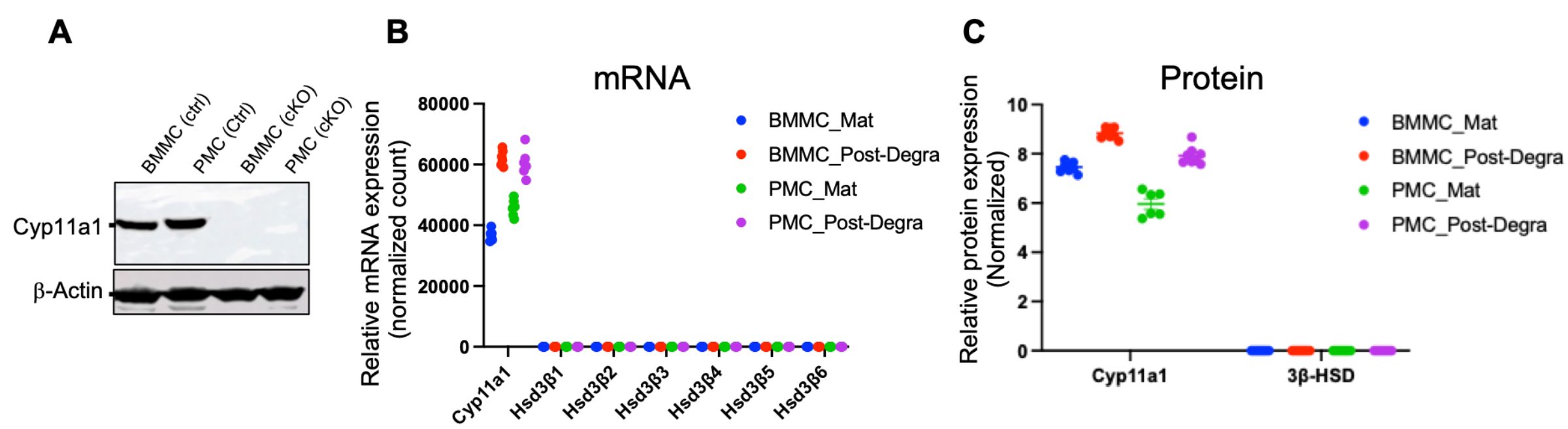

Fig. S2. (A) Western blot analysis confirming *Cyp11a1* deletion efficiency in BMMCs and PMCs derived from *Cyp11a1*$^{fl/fl}$;*Vav1*^{cre} (KO) and *Vav1*^{cre} control mice. β-Actin serves as loading control. **(B)** *Cyp11a1* and all isoform of 3β-HSD mRNA expression (*Hsd3β1*-6) in mast cells has been shown as revealed by RNA sequencing. **(C)** Cyp11a1 and 3β-HSD protein expression in mast cells has been shown as revealed by mass-spectrometry. (Mat: Mature; Post-Degra: Post-Degranulation)

Fig. S3

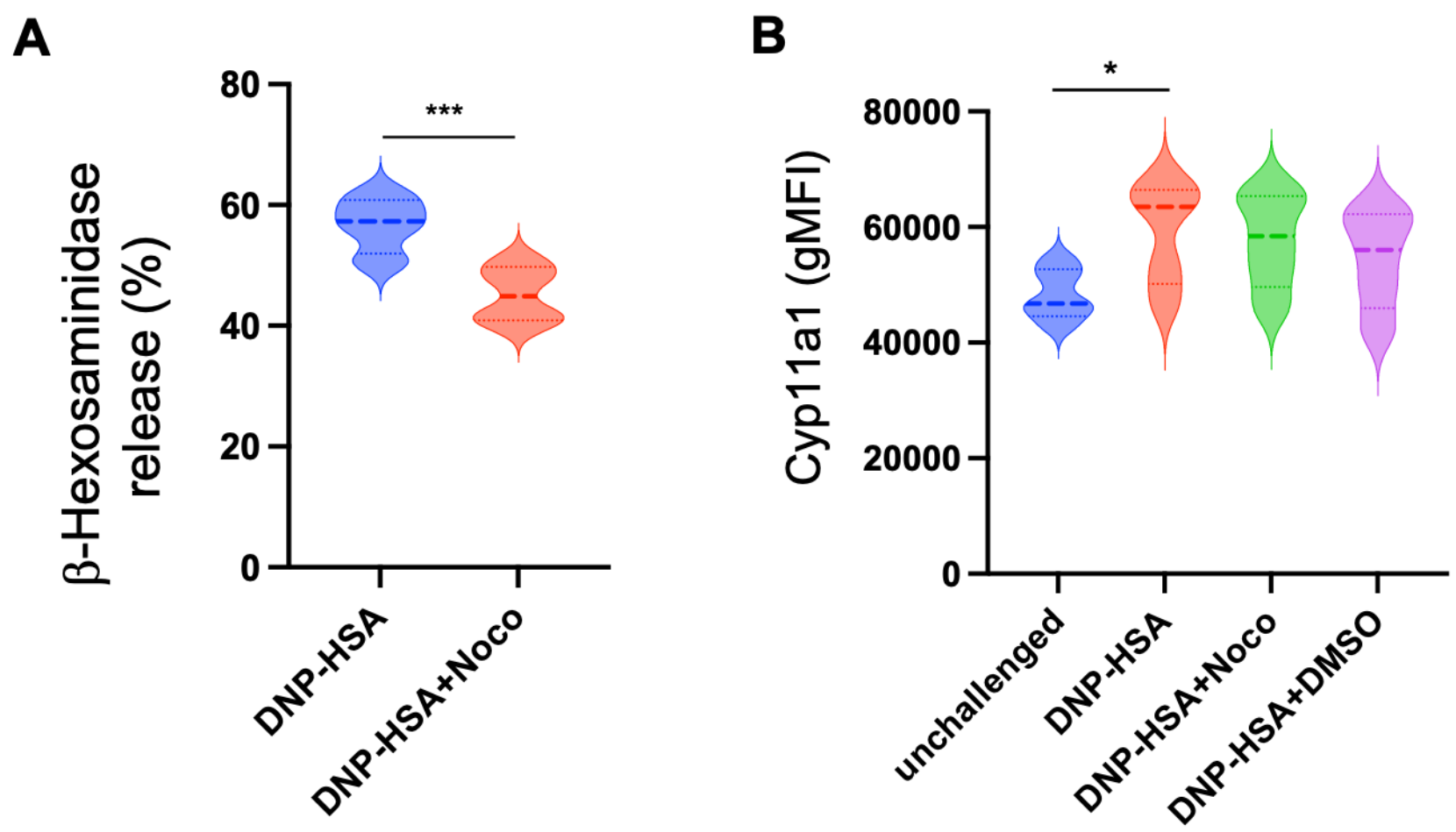

Fig. S3. Cyp11a1 upregulation is induced by mast cell activation independently of degranulation. (A) Nocodazole (10µg/ml, 33µM) inhibits IgE-mediated degranulation in peritoneal mast cells (PMCs). Anti-DNP IgE-sensitised PMCs were challenged with DNP-HSA in the presence or absence of nocodazole, and degranulation was quantified by β-hexosaminidase release. Violine plot shows that nocodazole significantly reduced β-hexosaminidase release. **(B)** Inhibition of degranulation does not prevent activation-induced Cyp11a1 expression. Cyp11a1 expression was measured in Cyp11a1-mCherry reporter PMCs by flow cytometry and is shown as geometric mean fluorescence intensity (gMFI). DNP-HSA stimulation increased Cyp11a1 expression relative to unchallenged cells, and this increase was maintained in the presence of nocodazole or DMSO vehicle. Data are representative of three independent experiments. Statistical significance was determined using paired two-tailed Student's t-test. ∗P<0.05, ∗∗∗P<0.001.

Fig. S4

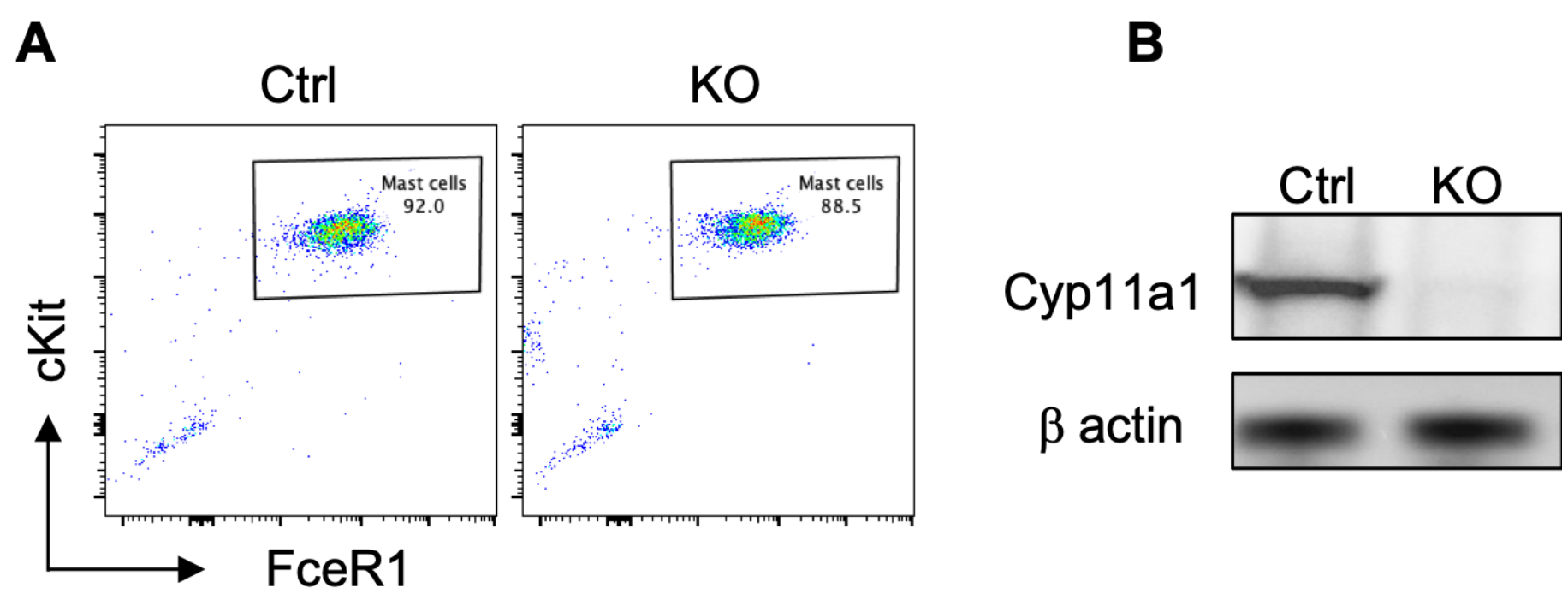

Fig. S4. Validation of mast cell differentiation and *Cyp11a1* deletion efficiency in *Cyp11a1*$^{fl/fl}$;*Cpa3*^{Cre} mice-derived PMCs. (A) Flow cytometric analysis of c-Kit and FceRI surface expression in control and mast cell-specific *Cyp11a1*-deficient PMCs. The gated c-Kit^{+}FceRI^{+} population indicates successful mast cell differentiation in both genotypes. Percentages indicate the frequency of mast cells within the gated population. **(B)** Immunoblot analysis of Cyp11a1 protein expression in cultured PMCs from control and *Cyp11a1* knockout mice, confirming efficient deletion of *Cyp11a1* in mast cells. β-actin serves as a loading control.

Fig. S5

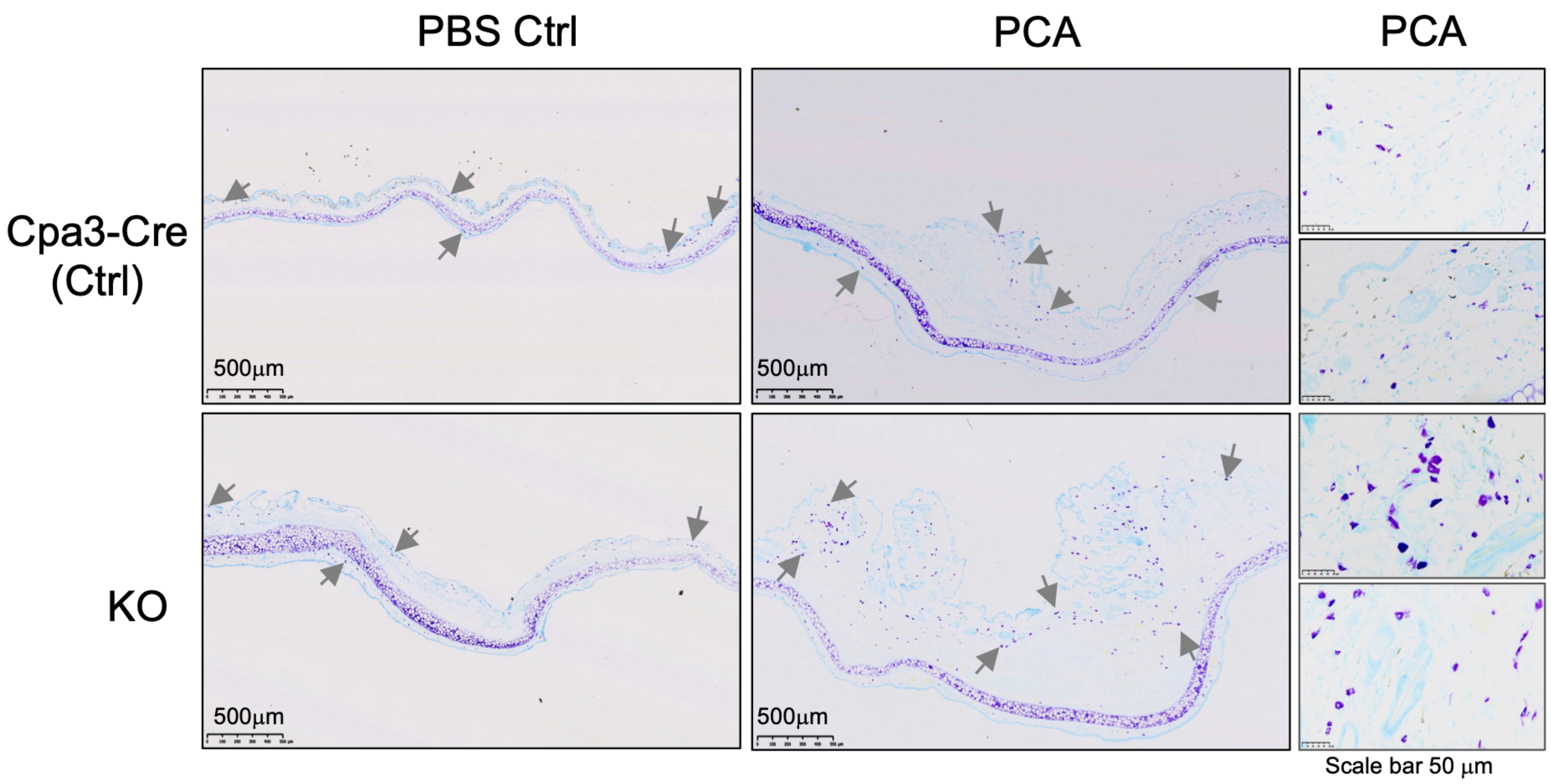

Fig. S5. Increased mast cell degranulation in Cyp11a1-deficient mice during passive cutaneous anaphylaxis.
Representative toluidine blue-stained ear sections from PBS- and PCA-treated control (*Cpa3*^{Cre}) and mast cell-specific *Cyp11a1*-deficient mice. Low-magnification images provide a broader view of the increased numbers of degranulating mast cells in Cyp11a1-deficient mice following PCA challenge compared with control mice. Arrows indicate representative mast cells in the tissue sections. Higher-magnification images of the PCA-treated groups are shown on the right. Scale bars, 500 µm in low-magnification images and 50 µm in high-magnification images.

Data S1-S10.

Supplementary Data S1. Differential gene expression in *Cyp11a1* knockout BMMCs. Differentially expressed genes comparing *Cyp11a1* knockout (KO) and control (*Cyp11a1*$^{fl/fl}$) bone marrow-derived mast cells (BMMC). This dataset was used to generate the volcano plot in Fig. 4C.

Supplementary Data S2. Differential gene expression in *Cyp11a1* knockout PMCs. Differentially expressed genes comparing *Cyp11a1* knockout (KO) and control (*Cyp11a1*$^{fl/fl}$) peritoneal mast cells (PMC). This dataset was used to generate the volcano plot in Fig. 4D.

Supplementary Data S3. Differential gene expression during post-degranulation recovery in BMMCs. Differentially expressed genes comparing in vitro generated BMMCs before degranulation and 8 h after degranulation. These genes are referred to as post-degranulation recovery associated genes. This dataset was used in Fig. 4L.

Supplementary Data S4. Differential gene expression during post-degranulation recovery in PMCs. Differentially expressed genes comparing in vitro generated PMCs before degranulation and 8 h after degranulation. These genes are referred to as post-degranulation recovery associated genes. This dataset was used in Fig. 4M.

Supplementary Data S5. Normalised expression of post-degranulation recovery-associated genes in BMMCs. Normalised expression levels across all experimental BMMC groups for the post-degranulation recovery associated genes identified in Supplementary Data S3. These data are presented in Fig. 4L.

Supplementary Data S6. Normalised expression of post-degranulation recovery associated genes in PMCs. Normalised expression levels across all experimental PMC groups for the post-degranulation recovery phase genes identified in Supplementary Data S4. These data are presented in Fig. 4M.

Supplementary Data S7. Differential protein abundance in female *Cyp11a1* knockout BMMCs. Differentially expressed proteins comparing *Cyp11a1* knockout (KO) and control (*Cyp11a1*$^{fl/fl}$) female BMMC. This dataset was used to generate the volcano plot in Fig. 5B.

Supplementary Data S8. Differential protein abundance in male *Cyp11a1* knockout BMMCs. Differentially expressed proteins comparing *Cyp11a1* knockout (KO) and control (*Cyp11a1*$^{fl/fl}$) male BMMC. This dataset was used to generate the volcano plot in Fig. 5C.

Supplementary Data S9. Differential protein abundance in female *Cyp11a1* knockout PMCs. Differentially expressed proteins comparing *Cyp11a1* knockout (KO) and control (*Cyp11a1*$^{fl/fl}$) female PMC. This dataset was used to generate the volcano plot in Fig. 5D.

Supplementary Data S10. Differential protein abundance in male *Cyp11a1* knockout PMCs. Differentially expressed proteins comparing *Cyp11a1* knockout (KO) and control (*Cyp11a1*$^{fl/fl}$) male PMC. This dataset was used to generate the volcano plot in Fig. 5E.
